# Ferroelectricity, Piezoelectricity, and Unprecedented Starry Ferroelastic Patterns in Organic–Inorganic (CH_3_C(NH_2_)_2_)_3_[Sb_2_X_9_] (X = Cl/Br/I) Hybrids

**DOI:** 10.1021/acs.inorgchem.5c00667

**Published:** 2025-05-05

**Authors:** Aleksandra Krupińska, Bogumiła Burzyńska, Vasyl Kinzhybalo, Błażej Dziuk, Przemysław Szklarz, Dariusz Kajewski, Jan K. Zaręba, Ada Drwęcka, Szymon J. Zelewski, Piotr Durlak, Piotr Zieliński, Paweł Sobieszczyk, Ryszard Jakubas, Anna Piecha-Bisiorek

**Affiliations:** † Faculty of Chemistry, University of Wrocław, F. Joliot-Curie 14, 50-383 Wrocław, Poland; ‡ Institute of Low Temperature and Structure Research, Polish Academy of Science, Okólna 2, 50-422 Wrocław, Poland; § Institute of Advanced Materials, Faculty of Chemistry, Wrocław University of Science and Technology, Wybrzeże Wyspiańskiego 27, 50-370 Wrocław, Poland; ∥ Institute of Physics, University of Silesia in Katowice, ul. 75 Pułku Piechoty 1, PL-41500 Chorzów, Poland; ⊥ Department of Experimental Physics, Faculty of Fundamental Problems of Technology, Wrocław University of Science and Technology, 50-370 Wrocław, Poland; # The H. Niewodniczański Institute of Nuclear Physics PAS, Radzikowskiego 152, Kraków 31-342, Poland

## Abstract

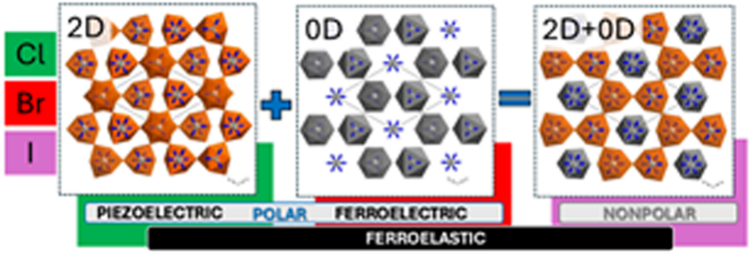

In this study, we present a novel class of lead-free hybrid antimony halides incorporating the acetamidinium cation, with the chemical compositions: (CH_3_C(NH_2_)_2_)_3_[Sb_2_Cl_9_] (**ACA**), (CH_3_C(NH_2_)_2_)_3_[Sb_2_Br_9_] (**ABA**), and (CH_3_C(NH_2_)_2_)_3_[Sb_2_I_9_] (**AIA**) . Despite their seemingly analogous chemical formulations, these compounds exhibit diverse physical characteristics, predominantly dictated by the differences in their metal-halide architectures. Indeed, the anionic frameworks of **ACA** and **AIA** are reminiscent of well-known ferroelectric materials, with **ACA** distinguished by its piezoelectric and ferroelastic characteristics, underpinned by a buckled honeycomb two-dimensional (2D) layers of antimony chloride. Conversely, **AIA** is characterized by its ferroelectric attribute, with discrete bioctahedral units forming a zero-dimensional (0D) structure. A surprising structural deviation constitutes the anionic sublattice of **ABA**, which amalgamates features from both **ACA** and **AIA**, yielding an unprecedented hybrid two-component (0D + 2D) anionic architecture. The ferroelectric properties of **AIA** have been demonstrated through pyroelectric current measurements and hysteresis loop analyses. Additionally, the noncentrosymmetric nature of **ACA** and **AIA** has been corroborated by second harmonic generation experiments. The piezoelectricity of **ACA** was confirmed using piezoresponse force microscopy (PFM). Furthermore, observations under a polarizing microscope revealed distinct ferroelastic properties in both **ACA** and **ABA**, characterized by well-defined and abundant star patterns previously observed only in simple oxides and alloys.

## Abstract 2

We present a novel class of lead-free hybrid antimony halides incorporating the acetamidinium cation, with the chemical compositions: (CH_3_C(NH_2_)_2_)_3_[Sb_2_Cl_9_] (**ACA**), (CH_3_C(NH_2_)_2_)_3_[Sb_2_Br_9_] (**ABA**), and (CH_3_C(NH_2_)_2_)_3_[Sb_2_I_9_] (**AIA**)

## Introduction

In recent years, organic–inorganic hybrid materials (OIHs) have emerged as a frontier class of compounds, garnering substantial scientific interest due to their exceptional versatility and potential applications across a broad spectrum of technological domains.^1−5^ These multifunctional materials have demonstrated remarkable promise in diverse fields, including gas storage, heterogeneous catalysis, chemical sensing, electronics, or photovoltaics. Among these applications, lead-based organic–inorganic halide perovskite solar cells have achieved particular prominence, exhibiting unprecedented theoretical power conversion efficiencies approaching 32%.^6−8^ Notwithstanding their impressive performance metrics, the widespread utilization of lead-containing hybrid materials presents significant challenges from both public health and environmental perspectives. The inherent toxicity of lead compounds, coupled with the susceptibility of lead-based hybrids to degradation under ambient conditions—including exposure to oxygen, moisture, elevated temperatures, and other environmental stressors—factors are the largest hurdles to commercialization and large-scale deployment.^9−11^ To resolve these critical concerns, various corrective measures can be taken, while preserving the desirable optoelectronic properties of these materials. One promising approach involves the partial or complete substitution of lead with less-toxic metal elements that possess matching physicochemical properties. For example, bismuth and antimony have emerged as particularly attractive candidates, which indicate similar electronic configuration and equivalent effective ionic radius as Pb.^12^ Of importance is also the fact, that Sb/Bi based OIHs exhibit a remarkable suite of optoelectronic and electrical properties, including ferroelectricity, piezoelectricity, and pyroelectricity, while simultaneously demonstrating good thermal stability.^13^ This unique combination of characteristics renders Sb/Bi based hybrids highly promising for a diverse array of technological applications. A distinctive feature of hybrids based on Bi^III^ and Sb^III^ halides, predominantly those with the chemical composition R_3_M_2_X_9_, is their propensity to form anionic subnetworks with varying dimensionalities. These structural motifs encompass zero-dimensional (0D) dimer structure,^14,15^ one-dimensional (1D) zigzag double chains,^16^ or two-dimensional (2D) layered architectures.^16^ The diversity in structural dimensionality plays a crucial role in modulating the physicochemical properties of these materials. In the specific case of Sb-based organic–inorganic perovskites incorporating the methylammonium (MA) cation (MA_3_Sb_2_I_9_), the anionic network is exclusively confined to 0D substructures within the hexagonal space group *P*6_3_/*mmc*.^13^ In this configuration, discrete 0D octahedral units [Sb_2_I_9_]^3–^ are encapsulated by the organic species. In turn, the purely inorganic analog, Cs_3_Sb_2_I_9_, exhibits remarkable structural polymorphism, capable of adopting both dimer (space group *P*6_3_/*mmc*) and layered 2D (space group *P*3̅*m*1) configurations, with the final structure heavily dependent on the specific synthetic methodology employed.^17^ The geometry and dimensionality of the anionic substructure exert a profound influence on the electrical properties of Sb-OIHs, particularly with respect to ferroelectricity. Notably, ferroelectric behavior in these systems is predominantly observed in 2D and 0D discrete bioctahedral units.^12^ This intriguing trend emerges when examining the relationship between structural dimensionality and halide composition: 2D structures are predominantly encountered in chloride and bromide ferroelectrics containing small alkylammonium moieties (such as methyl-, dimethyl-, and trimethylammonium) or nonsubstituted heterocyclic cations (*e.g*., pyrrolidinium).^18^ In stark contrast, all known iodide-based ferroelectrics invariably possess a 0D anionic network.^16,19−22^

The pursuit of novel, functional materials within the realm of OIHs is implemented on two interpenetrating levels. The first one concerns selecting the appropriate organic cation,^23^ while the second focuses on the modification of the anionic structure.^24−36^ In the latter aspect, the combination of diverse inorganic clusters within a single crystal lattice has emerged as an innovative approach to obtain, modify, or alter the macroscopic properties of the compound.^36−38^ It is noteworthy that all known two-component anionic structures are composed of discrete/isolated units linked by corners, edges, or faces. Recently, Wei et al. reported the discovery of two novel 0D molecular ferroelectrics based on the thiomorpholinium (TMP) cation, namely (TMP)_2_(SbX_5_)(SbX_3_) (X = Cl and X = Br).^39^ In these compounds, the anionic structure comprises discrete SbX_3_ and SbX_5_ polyhedrons separated by TMP cations. These OIHs exhibit remarkably high Curie temperatures (*T*_c_) (442/362 and 477/385 K for chloride and bromide analogs, respectively) and demonstrate giant zero-field entropy changes. Furthermore, they display significant room-temperature second harmonic generation (SHG) signals and polarization-dependent SHG responses. Ultraviolet–visible (UV–vis) absorption and photoluminescence spectra reveal a band gap shift from 3.366 to 2.600 eV, accompanied by a redshift in emission from 545 to 611 nm for Cl and Br, respectively.

In this study, we investigate the physicochemical properties of three cation-related OIHs: (CH_3_C(NH_2_)_2_)_3_[Sb_2_Cl_9_] (**ACA**), (CH_3_C(NH_2_)_2_)_3_[Sb_2_Br_9_] (**ABA**) and (CH_3_C(NH_2_)_2_)_3_[Sb_2_I_9_] (**AIA**). Despite their similar chemical compositions (R_3_M_2_X_9_ stoichiometry), these compounds exhibit diverse physical properties, primarily attributed to differences in their crystal structures. The construction of the anionic components in **ACA** and **AIA** are analogous to those observed in ferroelectric compounds, featuring a buckled honeycomb lattice of antimony chloride (2D layers) and discrete bioctahedral units (0D), respectively. Intriguingly, the anionic subnetwork geometry in **ABA** presents an unprecedented combination of inorganic parts observed in both **ACA** and **AIA**. At this point, it is imperative to emphasize that this type of mixed (two-component) anionic structure has been identified for the first time. Of special interest is the domain structure examined through optical polarization microscopy. All compounds undergo a proper ferroelastic phase transition (PT) with a reduction of 6-fold point symmetry. The resulting domain structure in **ABA** exhibits star patterns, observed in unprecedented abundance. The geometry of the domains and domain walls has been analyzed using group-theoretical method.^40^ A qualitative investigation of the impact of external stress on the domain composition in **ACA** allows one to hypothesize that the star patterns emerge post-PT when the sample can relax to form energetically most favorable nodes of domain walls. External and/or internal stresses either eliminate or reduce the occurrence of such nodes.

## Experimental Section

The X-ray diffraction (XRD) data were collected on an Oxford Diffraction Xcalibur four-circle diffractometer equipped with an Atlas CCD camera and a cryocooler device. The data were collected using Mo Kα radiation on: heating at 100, 295, and 365 K (**ACA**) and cooling at 305 and 100 K (**ABA**). Data reduction was carried out using CrysAlisPro (Rigaku Oxford Diffraction (2015); CrysAlisPro Software System, Version 1.171.42.90a. Rigaku Oxford Diffraction). Structure solution and refinement were carried out with the use of SHELXT^41^ and SHELXL.^42^ In **ABA**, low-temperature diffraction data revealed extensive twinning of the crystal, which is the result of the domain structure that appears on the transition from the hexagonal RT phase to the LT orthorhombic phase. Diffraction data for the LT phase were reduced as a six-component twin and refined with the use of a HKLF 5 type reflection file. Details on structure refinement are contained in deposited cif files. The powder diffraction patterns for the studied materials were collected in Bragg–Brentano geometry at room temperature on a PANalytical X’Pert Pro diffractometer using Cu Kα radiation and compared with the theoretical pattern simulated from RT single crystal data. Phase transition in **AIA** takes place on cooling from room temperature and is concerned with tripling of the *c*-axis (unique hexagonal axis). Tripling results in considerable reflections overlap (shown in reconstruction cuts of the Ewald sphere SI-Section 3), which disables the correct crystal system choice and consequently proper data reduction and structure solution of the low temperature phase. Room temperature phase is characterized by diffraction to low 2θ values what is most probably concerned with high disorder of organic cations and absorption. Therefore, the crystal structure of the room temperature phase of **AIA** is reported only as a model solved in *P*6_3_/*mmc* space group. It should be mentioned that the room temperature structure can also be solved in a number of hexagonal groups, so highest symmetry one was selected.

PFM measurements were performed in air with the use of the NanoWizard 3 Bio Science system by JPK Instruments, Berlin, Germany. The piezoresponse signal was analyzed on the polished surface of the crystal as the out-of-plane response at room temperature using soft platinum-coated cantilevers from MicroMash. The driving AC voltage used for measurements was 1 V at a frequency *f* = 10 kHz.

Nonlinear optical experiments were performed using a laser system employing a wavelength-tunable Topaz Prime Vis-NIR optical parametric amplifier (OPA) pumped by a Coherent Astrella Ti/Sapphire regenerative amplifier providing femtosecond laser pulses (800 nm, 75 fs) at a 1 kHz repetition rate. The measurements on **ACA** were performed using attenuated 800 nm output from a regenerative amplifier (laser fluence at sample of 0.25 mJ cm^–2^), while measurements of **ABA** and **AIA** were performed using the OPA output tuned to 1400 nm (laser fluence at a sample of 0.19 mJ cm^–2^). Single crystals of **ACA**, **ABA**, **AIA**, and KDP were crushed with a spatula and sieved through an Aldrich mini-sieve set, collecting a microcrystal size fraction of 88–125 μm. Next, size-graded samples were fixed in-between microscope glass slides to form tightly packed layers, sealed, and mounted to the horizontally aligned sample holder. No refractive index matching oil was used. The employed measurement setup operates in reflection mode. Specifically, the laser beam was directed onto the sample at 45 deg to its surface. Emission collecting optics consisted of a Ø25.0 mm plano-convex lens with a focal length of 25.4 mm mounted to the 400 μm 0.22 NA glass optical fiber and was placed along the normal to the sample surface. The distance between the collection lens and the sample was equal to 30 mm. The spectra of the temperature-dependent SHG responses were recorded by an Ocean Optics Flame T XR fiber-coupled CCD spectrograph with a 200 μm entrance slit. Scattered pumping radiation was suppressed with the use of a Thorlabs 750 nm hard-coated short-pass dielectric filter. Temperature control of the sample was performed using a Linkam LTS420 Heating/Cooling Stage. Temperature stability was equal to 0.1 K. TR-SHG study was conducted in a range of 293–368 and 113–293 K for **ACA** and **AIA**, respectively. The Kurtz-Perry powder test was performed by comparing the SHG signals of **ACA** (400 nm) and **AIA** (700 nm) collected at 293 and 123 K, respectively, with that of the KDP standard measured at 293 K for the corresponding wavelength, after normalizing SHG spectra to the same integration time.

Total energy calculations were performed using ab initio Density Functional Theory (DFT) as implemented in the CRYSTAL17^43^ package, designed for use in modeling crystalline solids. The calculations employed the London-type empirical correction in the (D3) variant for dispersion interactions as proposed by Grimme,^44−47^ including three-body dispersion contributions with fast analytical gradients together with the vibrational harmonic frequency calculations. The structural data (starting geometry) were taken from the X-ray crystal structure of **ACA** and **ABA** from the present study. The periodic *ab initio* calculations were performed utilizing the DFT-D3 methods with the range-separated (short-range corrected) hybrid functional, screened-Coulomb PBE functional combined with PBE correlation: HSE06-D3^48,49^ with the two shrinking factors (8′,8′) to generate a commensurate grid of *k*-points in reciprocal space, following the Monkhorst–Pack^50^ net method. All quantum-mechanical condensed matter simulations, including single point energy, geometric optimization of crystal structures and lattice parameters, electronic band structure (EBS), density of states (DOS) and piezoelectric tensor *d*_33_, were carried out with the consistent Gaussian basis sets of triple-ζ valence with polarization quality and BSSE-correction for solid-state calculations (pob_TZVP_rev2)^51−53^ proposed by Vilela-Oliveira, Peintinger, Laun, and Bredow in the second revision version. The electronic band structure was generated according to the procedure in the CRYSTAL17 program. The SeeK-path program was used to determine the *k*-points along a path within the first Brillouin zone, including the surface in reciprocal space.^54^ The EBS and DOS data from calculations were visualized a posteriori in the Gnuplot program (Williams, T., and Kelley, C. (2010). Gnuplot 4.4: an interactive plotting program). Exceptionally, piezoelectric tensor *d*_33_ was computed using the BLYP^55,56^ density functional because the method for generating these tensors in the Crystal17 program is not yet compatible with hybrid functionals such as HSE06-D3.

Photoacoustic spectra were measured in a setup utilizing microphone detection of acoustic waves generated inside a sealed custom-made cell through photoinduced material heating. The samples were excited with a monochromatic pumping (spectrally selected with a grating monochromator, Horiba iHR320) beam modulated mechanically at 10 Hz. Two light sources were used interchangeably depending on the spectral range, with a 250 W quartz-tungsten halogen (QTH) for photon energies <3 eV, and a 150 W xenon one above that. The signal proportional to the sound pressure inside the cell was demodulated with a lock-in amplifier (EG&G 7260), using the mechanical chopper output square wave signal as the reference.

Optical absorption spectra were measured using a tunable light source consisting of a 250 W QTH lamp and an Andor Kymera 328i. The light was mechanically chopped to allow lock-in detection (Stanford Research Systems SR860) of the analyzed signals from a silicon photodiode (Thorlabs FDS100) coupled to a transimpedance preamplifier (Thorlabs PDA200C) to convert the generated diode photocurrent to voltage.

The complex dielectric permittivity, ε* = ε′ – iε″, measurements were conducted on **ACA**, **ABA**, and **AIA** in the form of both single-crystal samples and polycrystalline pellets using an Agilent E4980A Precision LCR Meter between 100 and 370 K in the frequency range between 135 Hz and 2 MHz. Silver electrodes were painted on both opposite sides of the samples. The overall errors of ε′ and ε″ were less than 5%. The pyroelectric properties were tested with a Keithley 6517D electrometer/high resistance meter between 180 and 370 K, with a temperature ramp of 2 K min^–1^. Ferroelectric hysteresis loop measurements were carried out using a modified Sawer-Tower system with active electrical conductivity compensation and a Keysight InfiniVision DSO-X 2014A electronic oscilloscope with a 40 Hz triangular waveform generator. This signal was amplified using a Kepco BOP 1000 M high-voltage amplifier, from the output of which a driving voltage was applied to the sample. The sample was in a cooling system, regulated using a temperature controller from Shimaden, model FP23. The system was controlled using proprietary computer software. To compensate for electrical conductivity, the compensation current was set at room temperature to obtain a straight line in the *P*–*E* relationship, inclined to the *E* axis. Then, the sample was cooled to −120 °C (c.a. 153 K), and ferroelectric hysteresis loops’ measurements were carried out with increasing driving fields from 1.5 to 12 kV cm^–1^.

The piezoelectric charge coefficient (*d*_33_) of **ACA** was measured on a single crystal sample along the *c*-direction using the quasistatic (Berlincourt) method. An APC International wide-range *d*_33_ tester, operating with a force-frequency of 110 Hz and an amplitude of 0.25 N, was employed. The electrical contacts were made with silver conductive paste.

The formation of ferroelastic domains was recorded under polarized light using an Olympus BX53 microscope. The temperature change was operated with a LINKAM THM-600 heating/cooling stage, which allowed temperature stabilization of 0.1 K.

## Results and Discussion

(CH_3_C(NH_2_)_2_)_3_[Sb_2_Cl_9_] (**ACA**) and (CH_3_C(NH_2_)_2_)_3_[Sb_2_Br_9_] (**ABA**) were synthesized from Sb_2_O_3_ and acetamidinium chloride/bromide in HCl and HBr solution, respectively. In turn (CH_3_C(NH_2_)_2_)_3_[Sb_2_I_9_] (**AIA**) was synthesized from SbI_3_ and acetamidinium iodide in HI solution (more information in Supporting Information – Section 1). After heating the precursor solutions to 90 °C for 20 min, followed by controlled evaporation at ambient temperature, resulting in the formation of single crystals: colorless for **ACA**, yellow for **ABA**, and red for **AIA**, all stable at room temperature, were formed (Figure S1). Details on synthesis protocols, purity (Figure S2), and composition (Table S1) are provided in the SI. All compounds undergo one reversible PT (Figure S3) at 345 K for **ACA**, 292 K for **ABA**, and 278/275 (heating/cooling) K for **AIA**. These materials also exhibit substantial thermal stability with decomposition temperatures of 410, 500, and 470 K, respectively (Figure S4).

### X-ray Structure Analysis

**Figure 1:**
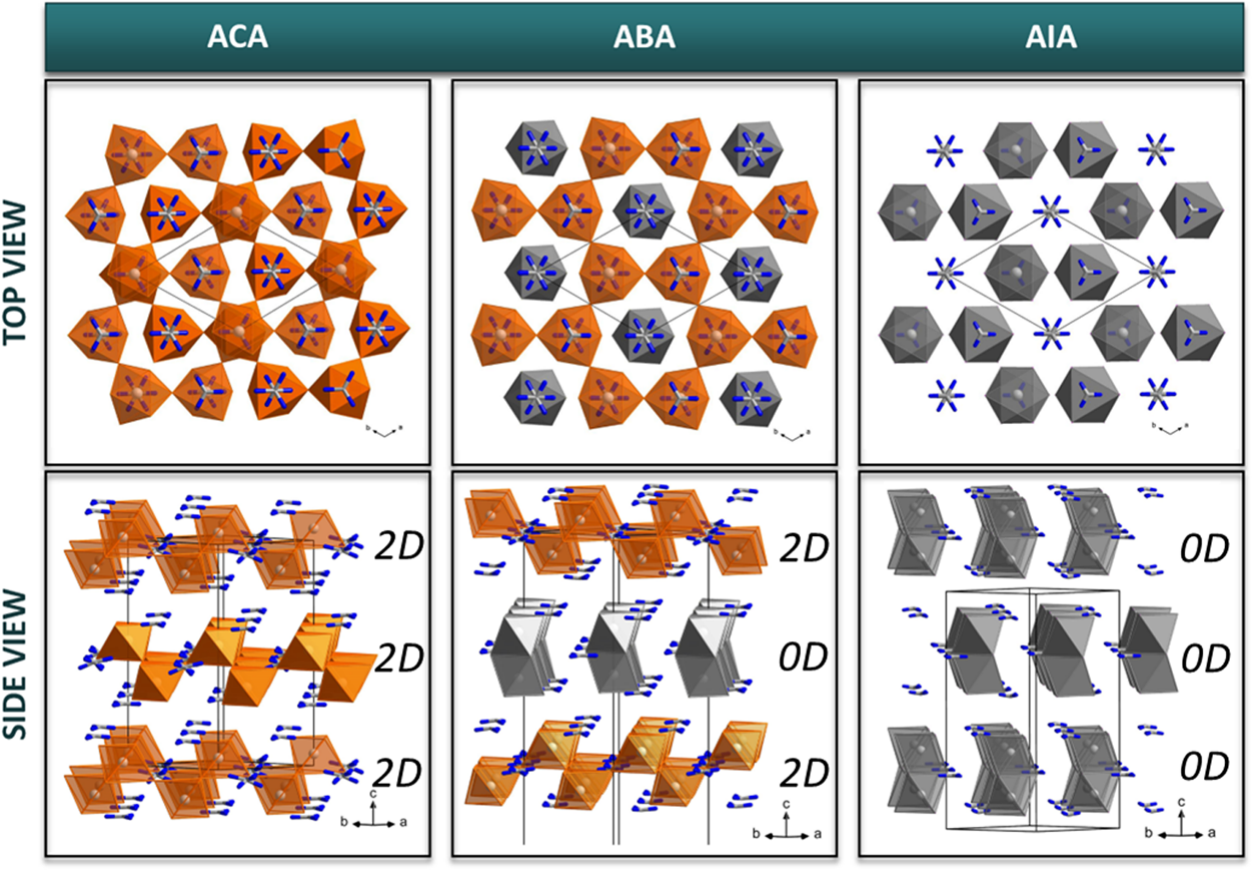
Comparison of the crystal packing for **ACA**, **ABA**, and **AIA**. Orange and gray octahedra belong to 2D infinite layers and 0D bioctahedra, respectively

X-ray diffraction studies revealed significant structural diversity within this series of compounds, making a rare example of a chloride-bromide-iodide progression with nonisomorphous crystal structures. The anionic substructure of **ACA** is purely two-dimensional (2D), while **AIA** features zero-dimensional (0D) dimeric anions. **ABA** displays a unique hybrid structure incorporating elements from both chloride and iodide analogs (Figure 1). The SI presents detailed structural descriptions of each phase of **ACA**, **ABA**, and **AIA** (Section 3).

**Figure 2:**
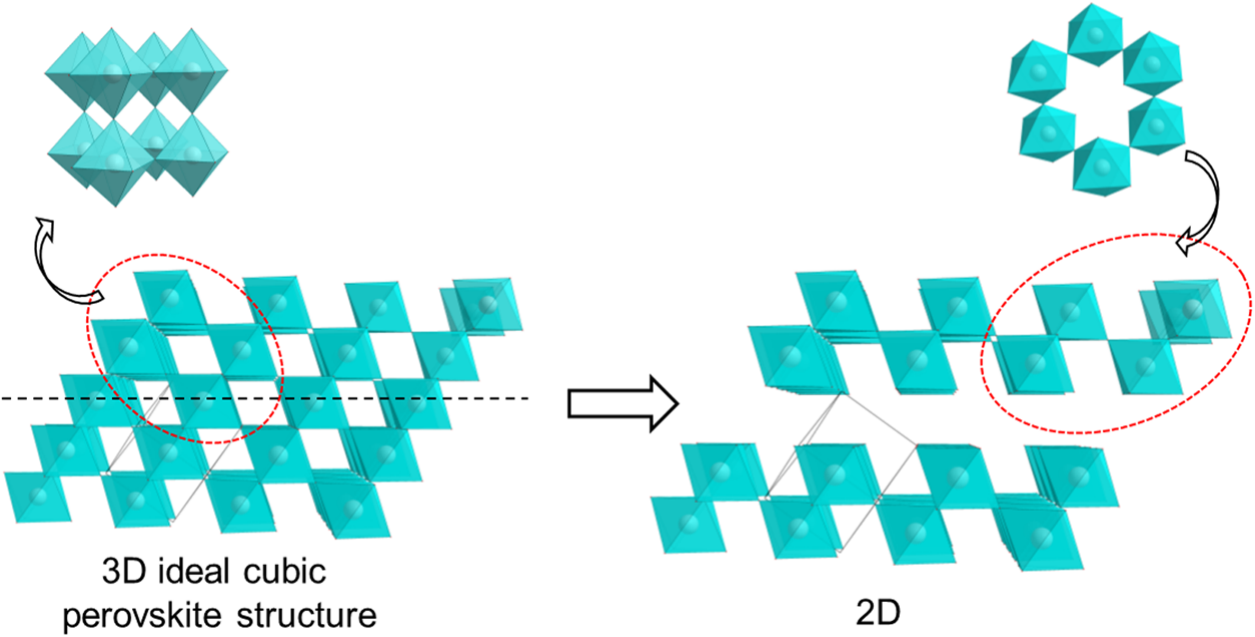
[Sb_2_X_9_]*_n_*^3*n*–^ 2D layer as a part of cubic perovskite framework (cations are omitted for clarity).

The layers in **ACA** are built of connected into a 2D network of [SbCl_6_] octahedra with three terminal and three bridging halogen Cl ions that occupy opposite octahedron faces. The [Sb_2_Cl_9_]^3–^ layer can be conceptualized as a 2D perovskite-type structure derivable from an ideal cubic perovskite through removal of every third Sb-layer along the (111) direction (Figure 2). While octahedra occupy two levels within the layer, cations are distributed across three levels: two external and one internal. The internal positions are located at the centers of hexagonal cavities, while external positions are directly above or below the [SbCl_6_] octahedra at the centers of triangles formed by top or bottom octahedra (Figure 1). Interestingly, the cation positions in the [Sb_2_Cl_9_]^3–^ layer are consistent with the ones in the parent perovskite structure. The overall crystal packing in **ACA** follows an ···2D-2D-2D··· stacking sequence with antiparallel neighboring layers (Figure 1). The crystal packing in **AIA** is governed by the formation of [Sb_2_I_9_]^3–^ bioctahedra, that share a common face. The bioctahedra are arranged in a triangular lattice. Similarly to **ACA**, the layers in **AIA** produce ···0D-0D-0D··· crystal packing with antiparallel neighboring layers (Figure 1).

In turn, crystal packing in **ABA** (Figure 1) is governed by the presence of both layers (as in **ACA** orange) and bioctahedra (as in **AIA-**gray) that pack alternately (···2D-0D-2D···) which create an exceptional two-component anionic substructure observed, for the first time, among OIHs.

The acetamidinium cations play a consistent structural role across all the compounds examined, exhibiting free in-plane rotation within in HTPs. Despite the structural differences observed among the compounds, their characteristics result in crystallization into high-symmetry hexagonal structures, which transform to lower symmetry upon cooling. Although the LTP crystal structure of **AIA** could not be solved, it is evident that symmetry lowering occurs upon cooling (see Figure S8). This symmetry reduction in all reported substances is attributed to the restriction of acetamidinium rotation. The formation of relatively strong N–H···X hydrogen bonds between the cationic and anionic substructures facilitates the long-range transmission of cation ordering information (more in SI – Section 3) (Table 1).

**Table 1:** Experimental Data for **ACA**, **ABA** and **AIA**

crystal data						
abbreviation	**ACA**			**ABA**		**AIA**
empirical formula	C_6_H_21_N_6_Sb_2_Cl_9_			C_6_H_21_N_6_Sb_2_Br_9_		C_6_H_21_N_6_Sb_2_I_9_
formula weight (g·mol^–1^)	739.84			1139.98		1562.89
crystal system	orthorhombic		hexagonal	orthorhombic	hexagonal	hexagonal
space group	*Cmc*2_1_		*P*6_3_*mc*	*Cmcm*	*P*6_3_/*mmc*	*P*6_3_/*mmc*
temperature (K)	100	295	365	100	305	290
unit cell dimensions						
*a* (Å)	8.476(3)	8.758(3)	8.940(3)	8.630(3)	9.137(4)	9.3310(16)
*b* (Å)	16.087(5)	15.818(5)		16.718(4)		
*c* (Å)	17.690(6)	18.375(6)	18.811(5)	37.493(7)	39.705(7)	22.122(4)
*V* (Å^3^)	2412.2(14)	2545.8(14)	1302.2(9)	5409(2)	2870(3)	1668.1(7)
*Z*	4	4	6	8	4	2
*D*_calc_ (g·cm^–3^)	2.04	1.93	1.89	2.80	2.64	3.11
μ (mm^–1^)	3.24	3.07	3.00	15.30	14.42	9.95
*F*(000)	1416	1416	708	4128	2064	1356
crystal size	0.31 × 0.16 × 0.07			0.21 × 0.13 × 0.04		0.19 × 0.12 × 0.03
diffractometer	Xcalibur, Atlas					
monochromator	Graphite Mo Kα					
radiation type, wavelength λ (Å)	Mo Kα, 0.71073					
absorption correction	analytical, *CrysAlis PRO* 1.171.41.80a (Rigaku Oxford Diffraction, 2020) empirical absorption correction using spherical harmonics, implemented in SCALE3 ABSPACK scaling algorithm.					
reflections collected/independent/observed, [*R*(int)]	19,971, 3285, 3231, 0.026	19,980, 3431, 2628, 0.035	4858, 1062, 499, 0.049	24,355, 24,355, 12714, - (twin)	20,528, 1533, 593, 0.095	4553, 832, 177, 0.195
parameters	125	143	58	105	59	22
goodness-of-fit on *F*^2^	1.09	1.02	1.00	1.05	1.04	1.06
*R*[*F*^2^ > 2σ(*F*^2^)], *w*R**(*F*^2^)	0.015, 0.031	0.032, 0.066	0.033, 0.091	0.086, 0.309	0.076, 0.239	0.148, 0.308
Δρ_max_, Δρ_min_ (e Å^–3^)	0.48, −0.32	0.47, −0.34	0.31, −0.36	3.24, −3.80	0.89, −0.44	0.77, −0.60
CCDC	2400844	2327666	2327667	2400843	2400842	

### Second Harmonic Generation Studies

**Figure 3:**
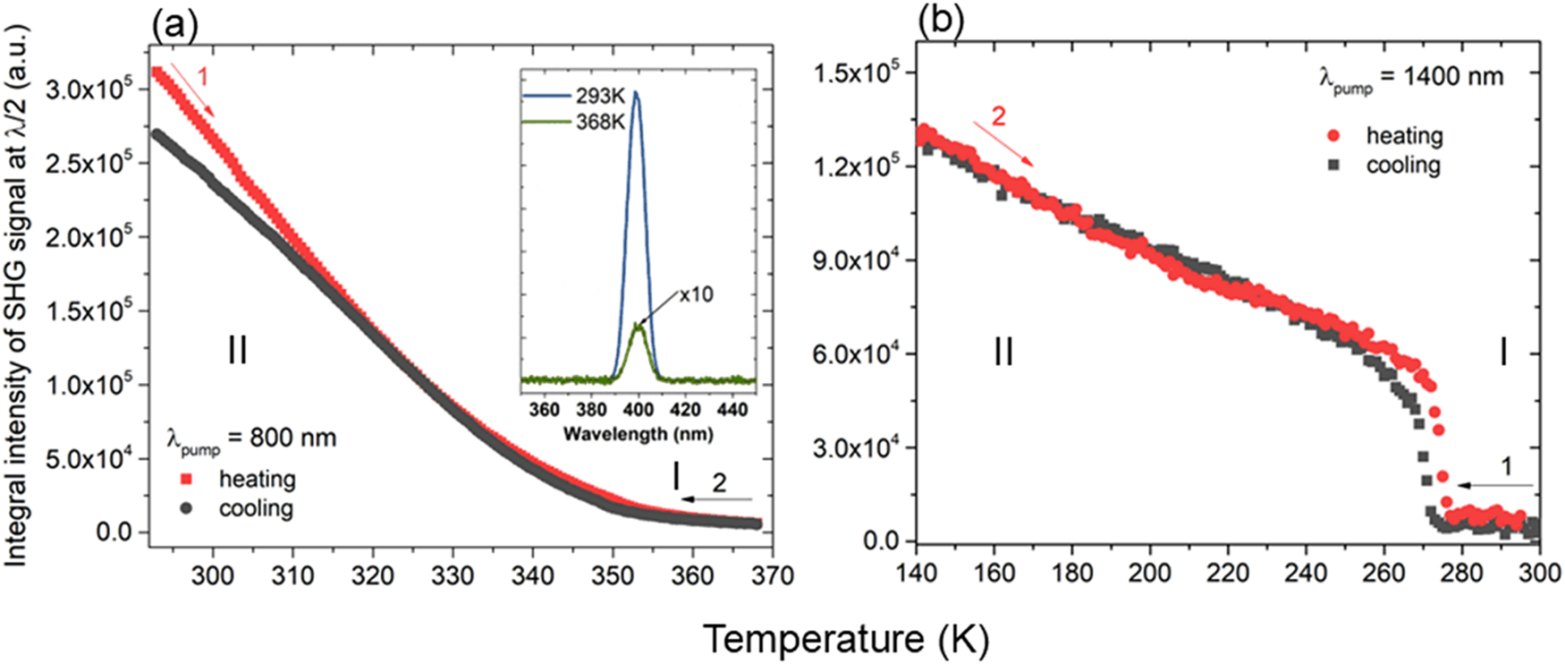
Temperature plots of SHG integral intensities for (a) **ACA** (the inset shows a comparison of SHG traces of **ACA** captured at 293 and 368 K upon 800 nm irradiation) and (b) **AIA**.

Our studies further extended into temperature-resolved second harmonic generation (TR-SHG) measurements of the title compounds. TR-SHG studies of **ACA** were carried out across a temperature range of 293–368 K utilizing 800 nm femtosecond laser pumping. The temperature plot of SHG intensities (λ_2ω_ = 400 nm) is depicted in Figure 3(a), with the corresponding SHG spectral data provided in Figure S9(a,b). The data in Figure 3(a) shows the presence of an SHG response at room temperature, which decreases monotonically with increasing temperature. The absence of the temperature hysteresis along with the continuous change of SHG intensity suggests the second-order character of the PT between phases I and II. Nevertheless, the SHG data at the high-temperature and corresponding to phase I requires a comment. Namely, one sees that the SHG intensity significantly decreases with temperature to the point that near 368 K where the strength of this response may seem to approach zero. Inspection of experimental spectra shows this is not the case here (see inset in Figure 3(a)). Indeed, if we relate the SHG intensity vs that of KDP standard (Kurtz-Perry powder test) we find a relative SHG efficiency of 1.31 at 293 K (Figure S10(a)) and a moderate value of 0.02 at 368 K. It follows that already strong SHG of **ACA** at RT, significantly decreases but does not disappear with temperature. Thus, the presence of SHG across the studied temperature range supports the assignment of a noncentrosymmetric setting for both phases I and II. The SHG screening of **ABA** with 1400 nm femtosecond laser pumping in 93–293 K range did not reveal any second harmonic response, corroborating the assignment of centrosymmetric space groups to crystal phases by SCXRD.

TR-SHG results collected for **AIA** in 113–293 K range and 1400 nm femtosecond laser pumping are provided in Figure 3(b) (experimental traces in Figure S9(c,d)). In this case is observed a steep increase in SHG signal intensity (λ_2ω_ = 700 nm) upon cooling at ca. 275 K, with a loss of SHG activity upon heating beyond 278 K. This reflects symmetry breaking and restoration, respectively, indicating that the PT occurs between noncentrosymmetric (phase II) and centrosymmetric (phase I) structures. The presence of an approximately 3 K-wide temperature hysteresis indicates a first-order character of this PT, in agreement with DSC and dielectric results. In the low-temperature region, one observes a systematic increase of SHG activity, yet without noticeable anomalies. The Kurtz-Perry test of **AIA** at 1400 nm shows a moderate SHG intensity equivalent to 0.046 times that of KDP (Figure S10(b)).

### Dielectric Properties

**Figure 4:**
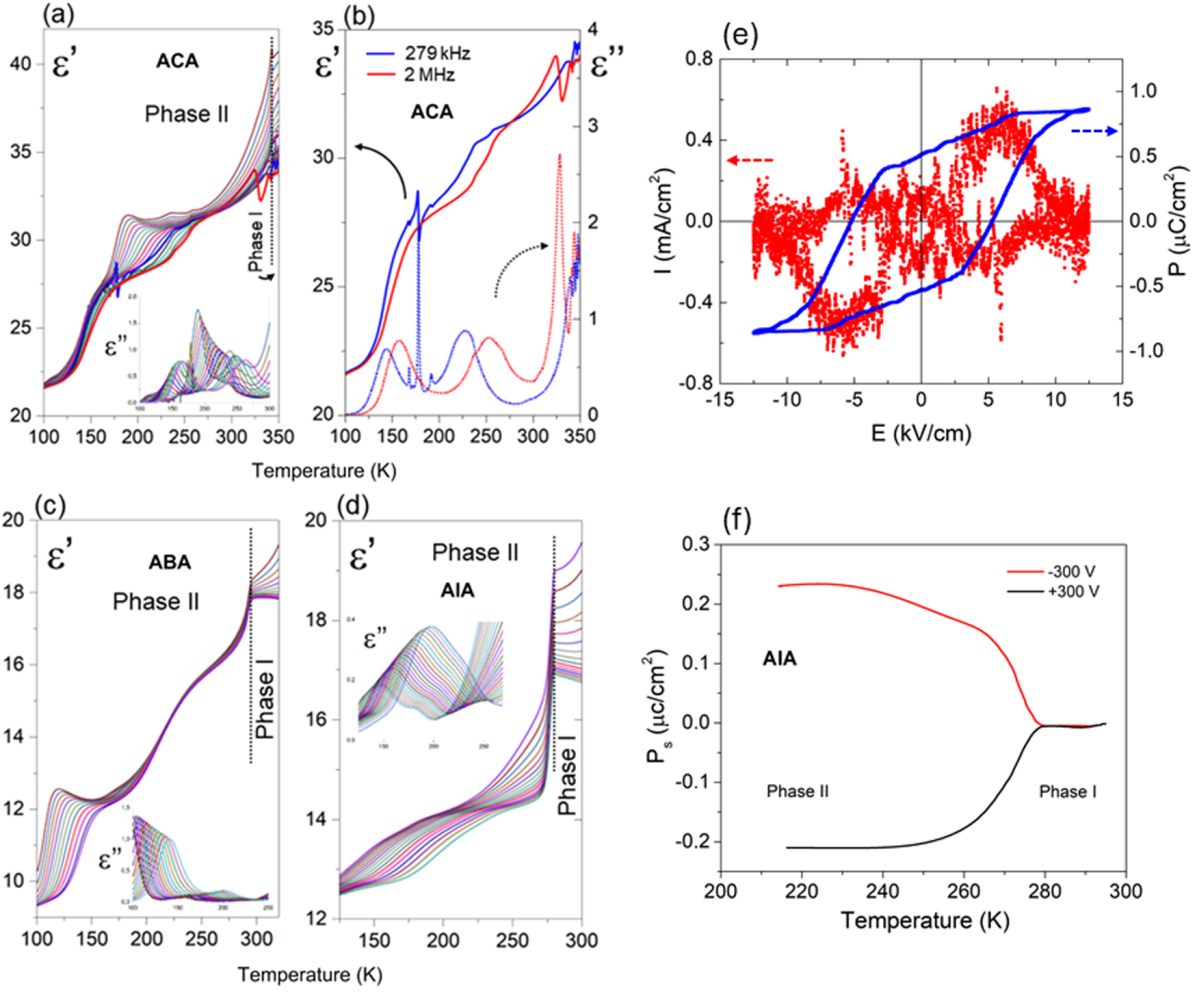
Dielectric response of the **ACA** (a, b), **ABA** (c), and **AIA** (d) at different frequencies (135 Hz–2 MHz) as a function of temperature. Panel (b) shows resonant piezoelectric contributions observed in **ACA**. Insets in Figures (a, c, d) illustrate the ε″ vs temperature (relaxation process); (e) Measured (*I*–*E*) and calculated (*P*–*E*) hysteresis loop at 220 K; (f) Temperature changes of the spontaneous polarization of **AIA** after pooling by an external electric field.

Dielectric measurements were performed at different frequencies on a single-crystal **ACA** (along the *c*-axis) and pellet samples of **ABA**, and **AIA** within the temperature range of 100 to 355 K (refer to Section 5 in SI). The plot of the a real and imaginary part of complex dielectric permittivity measured as a function of temperature is presented in Figure 4(a–d). In the case of **ACA** (Figure 4(a)), the structural anomaly from polar phase I (*Cmc*2_1_) to polar phase II (*P*6_3_*mc*) is marked by a slight increase in electric permittivity when approaching *T*_*c.*_ In contrast, for the remaining two (**ABA** and **AIA**), a distinct step-like anomaly in their electric response (ε′) curves are observed (Figure 4(c),(d)). Moreover, in the case of **AIA** (Figure 4(d)), in the vicinity of the PT (in phase II), the dielectric anomalies at lower frequencies are not so sharp. This is probably, due to the presence of the ferroelectric domain walls affecting electric permittivity over phase II. Additionally, **ACA** displays characteristic resonance curves in the ε′(*T*) dependence at experimental frequencies of 279 kHz and 2 MHz, along with related absorption anomalies for the **ACA** sample are typical of crystals lacking a center of symmetry (Figure 4(b)). These are attributed to piezoelectric resonances contributing to electric permittivity. The piezoelectric tensor (*d*_33_) component for **ACA** was calculated using an analytical approach based on the CPHF/KS scheme. The calculated value of the piezoelectric tensor equals 6.0 pC N^–1^ which is in good agreement with the experimental results (*d*_33_ = 6.3 pC N^–1^).

**Figure 5:**
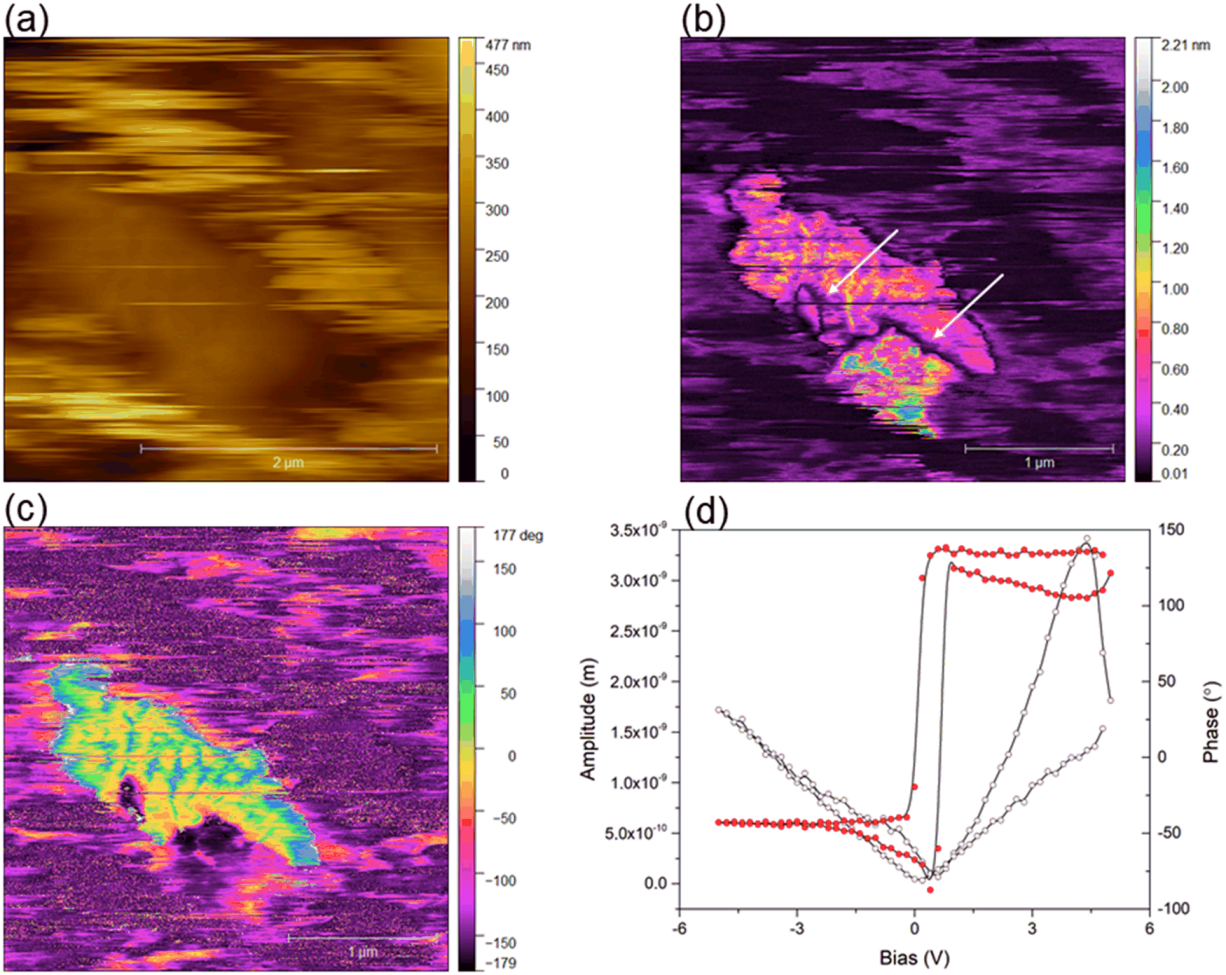
(a) Topography, (b) amplitude, (c) phase of out-of-plane piezodeformation signal obtained by atomic force microscopy; (d) The PFM amplitude-bias and phase-bias ‘butterfly’ and ‘hysteresis’ loop of **ACA**.

We also conducted pyroelectric and hysteresis loop measurements to assess the polar (ferroelectric) properties of **ACA** and **AIA** crystals. For **ACA**, we were not able to detect the ferroelectric hysteresis loop, which indicates the piezoelectric (nonferroelectric) character of both phases. Conversely, **AIA** demonstrated the ferroelectric properties of phase II were proved by the hysteresis loop measurement (Figure 4(e)). The reversibility of spontaneous polarization (*P*_s_) was confirmed through pyroelectric effect measurement in which the sample was polarized by an external electric field, *E*_ext_, as high as ±3 kV cm^–1^ in phase I and then gradually cooled down to phase II. After removing the external field, the sample was shorted for several minutes. Figure 4(f) illustrates the change in *P*_s_ during the heating cycle, indicating a decrease. The result indicates that the *P*_s_ value diminishes in the discontinuous PT. The change in the *P*_s_ value found in **AIA** equals ca. 0.2 μC cm^–2^. To verify the piezoelectric properties in **ACA** the PFM measurements were undertaken. Figure 5 presents scans of topography (a), the amplitude of out-of-plane piezodeformation (b) and phase (c) for **ACA**. Despite polishing, some surface contamination caused ‘noise’ in the topography image. However, in clear surface areas, significant piezodeformation was observed (Figure 5(b) – bright areas), alongside regions with no deformation (black) and weak deformation (violet). Within the bright regions showing strong piezodeformation some domain walls could be observed (marked with arrows in Figure 5(b)) which are 180° domain walls, since the observed phase changes between the two neighboring domains are about 180° (Figure 5(c)). This indicates that these domains have polarization perpendicular to the crystal surface, but the directions of the vector are opposite. In this region, a so-called butterfly loop with a very small coercive field was obtained (Figure 5(d)), confirming that **ACA** exhibits piezoelectric properties at least on a microscale.

In this group of compounds, low-frequency relaxation processes related to the dynamics of acetamidinium cations are evident in phase II (Figure 4(a),(c),(d)). For **ACA** (Figure 4(a)), two distinct low-frequency relaxation processes can be observed between 100 and 180 K (relaxator 1) and 180–325 K (relaxator 2). These relaxators exhibit fundamentally different dynamic characteristics. Relaxator 1 is characteristic of materials described by the Vogel–Fulcher relation, while relaxator 2 is indicative of critical slowing down, typical in ferroelectric crystals. In **ABA** (Figure 4(c)), two relaxators can also be distinguished. Relaxator 1 between 100 and 175 K resembles relaxator 2 in **ACA** but with less pronounced slowing down. In turn, relaxator 2 (in the temperature range of 175–230 K) is characterized by a tiny increment (approximately 0.15). It should be underlined that the Cole–Cole relation can describe the relaxation processes in the case of **ABA**. Regarding **AIA** (Figure 4(d)), a single polydisperse relaxation process is noted between 130 and 230 K, which can be described by the Vogel–Fulcher relation, characteristic of ferroelectric relaxors.^57^

The results obtained from dielectric measurements, especially when compared with SHG and structural analysis, shed new light on the physicochemical properties of **ACA** and **AIA**. The anionic structure of **ACA** is typical of ferroelectric crystals with the R_3_M_2_X_9_ composition, featuring two-dimensional anionic layers. It also includes two types of small, mobile acetamidinium counterions in the crystal structure (within (type B) and between (types A and C) layers), thus from the structural point of view, all criteria are met to obtain a ferroelectric crystal. Both phases are described by noncentrosymmetric/polar space groups (*P*6_3_*mc* for phase I and *Cmc*2_1_ for phase II), as confirmed by SHG experiments, while PFM analysis verified piezoelectric properties. Surprisingly, ferroelectric hysteresis loops were not detected in either phase, leaving ferroelectric properties of **ACA** unconfirmed.

In our opinion, the absence of ferroelectricity in **ACA** is conditioned mainly by the arrangement of the inorganic sublattice arrangement. The experimental results, collected so far, suggested that the origin of ferroelectricity in the R_3_M_2_X_9_-type of compounds is connected strictly with the dynamics of organic cations within vacancies created by a two-dimensional inorganic sublattice. The dynamics of cations occupying 12-membered, strongly distorted rings within the layers exhibit critical slowing down as they approach the paraelectric-ferroelectric PT, contributing to spontaneous polarization and long-range dipole order. Typically, this ferroelectric transition is usually accompanied by a drastic distortion of the 12-membered rings in the inorganic sublattice. On the other hand, in the R_3_M_2_X_9_-ferroelectrics inorganic layers are stacked with a moderate shift giving rise to channels occupied by organic cations. Such an arrangement enables a long-range ordering of polar cations in the ferroelectric phase.

In **ACA**, the dynamics of acetamidinium cations differ as all of them are disordered at high temperatures and become ordered at room temperature, except for cation B (within 12-membered rings), which remains disordered in two equally occupied positions around mirror planes. Furthermore, the inorganic layers are mutually shifted, so that below and above each vacancy of the 12-membered net one can find the SbCl_6_ octahedra of adjacent inorganic fragments This arrangement likely screens dipolar interactions between organic cations, hindering the long-range ordering necessary for a ferroelectric phase occurrence.

In turn, the ferroelectric properties of the **AIA** have been confirmed through pyroelectric current measurements and observation of ferroelectric hysteresis loops in phase II. However, without single-crystal structure analysis, we cannot fully explain the molecular mechanism behind this paraelectric-ferroelectric transition. However, taking into account the estimated value of entropy of this transformation (Δ*S*_(I–II)_ = 42.6 J mol^–1^ K^–1^) detected calorimetrically, it is likely that the dynamics of acetamidinium cations contribute, for the most part, to the PT mechanism.

### Solid State Static Calculation

**Figure 6:**
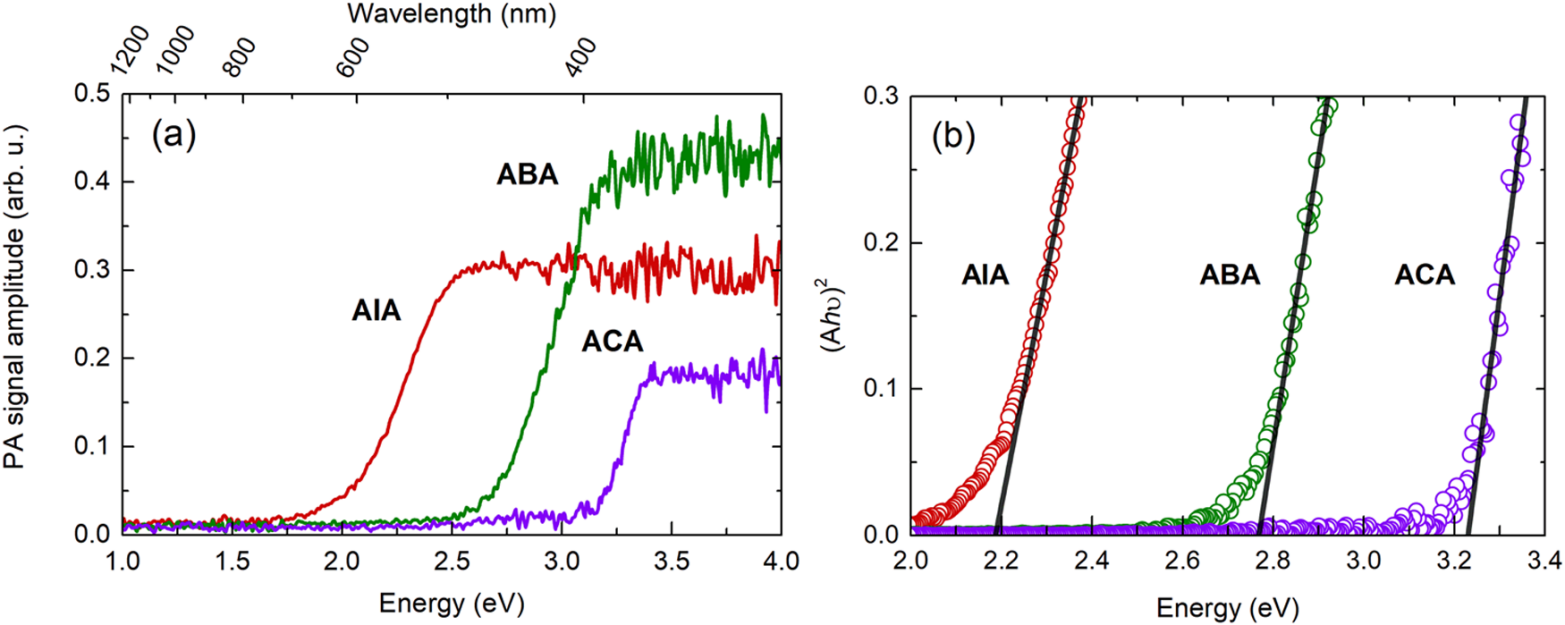
(a) Photoacoustic absorption spectra obtained for **AIA**, **ABA**, and **ACA**, (b) Tauc plots for each compound used for determination of band gap energies.

The optimization of geometric and structural parameters for the crystal structure of **ACA** and **ABA** carried out at the DFT, demonstrates a strong correlation between the calculated geometric parameters (including bond lengths, angles, dihedral angles, and lattice parameters) and experimental data (see Tables S8 and S9). The deviation in optimized structural parameters does not exceed 10%. As illustrated in Figures S12 and S13, **ACA** and **ABA** exhibit a wide band gap of approximately 3.64 and 3.00 eV, respectively, and DOS calculations suggest semiconductor or insulator characteristics. The densities of states projected onto the energy band diagram show that the highest valence states are predominantly composed of Cl(p) states, whereas the lowest conduction band states predominantly involve Sb(p) states, (Figures S12 and S13). The alignment of the conduction band minimum and valence band maximum at the same crystal momentum (*k*-vector) in the Brillouin zone (the Γ point) indicates a quasi-direct band gap.^58−60^ We further investigated the band structure experimentally through photoacoustic spectroscopy on all three compounds. Photoacoustic detection, relying on indirect sensing of optical absorbance through conversion of light energy to heat, was chosen due to apparent issues with spectra obtained using transmission mode measurements (Figure S14). A strong baseline below the main absorption edge, likely originating from light scattering and suboptimal sample thickness-light extinction relation, makes such experiments insufficient for reliable characterization of bulk crystals. The spectral traces depicted in Figure 6(a) are assumed to follow a linear relationship with the optical absorption coefficient (α) for energies below the band gap, with the bottom detection limit set by the defect-related absorption of the crystal or, in this case, the sensitivity of the experimental setup.

Characteristic signal saturation occurs upon reaching a regime where the optical penetration depth (1/α) becomes smaller than the thermal diffusion length, defined by the thermal properties of the material.^61^ Absorption edges for **AIA** and **ABA** lie within the visible range, while **ACA** remains optically transparent up to about 3.1 eV (400 nm), which coincides with the translucent appearance of the studied crystals (see Figure S1(a)). The observed absorption blue shifts upon replacing heavier halogens with lighter ones in the inorganic cage, which is typically observed for hybrid compounds.^61−63^ Band gap energies were determined for all materials using the Tauc plot analysis (substituting the classically used α with the signal amplitude, *A* in the subgap range), assuming direct allowed optical transition. Their values span from 2.18 eV for **AIA**, through 2.77 eV for **ABA**, up to 3.23 eV for **ACA** (Figure 6(b)). The experimental result therefore confirms the high band gap predicted theoretically from *ab initio* calculations. The measured absorption edges are considerably blueshifted compared to 3D hybrid halide perovskites, characteristic of reduced dimensionality of the structure.^64^

### Ferroelastic Domains in Orthorhombic Phase

The PTs in the compounds **ABA**, **ACA** and **AIA** at *T* = 292, 345, and 275/278 K respectively, reduce the initial hexagonal symmetry to an orthorhombic symmetry. One of the orthorhombic axes stays parallel to the initial 6-fold axis. This hypothesis is maintained for **AIA** despite challenges in determining its crystallographic system in LT phase. The space group – subgroup relationship for **ABA** is *P*6_3_/*mmc* → *Cmcm* and for **ACA***P*6_3_*mc* → *Cmc*2_1_. In both cases, the number of atoms per primitive cell remains constant during the PT because the orthorhombic space groups are base-centered, meaning there is no reduction in translational symmetry. Consequently, we deal here with proper ferroic species. The Aizu classification of ferroics^65^ allows one to summarize the changes of point groups. For **ABA** the species is 6*/mmmFmmm* and for **ACA** 6*mmFmm*2. The lowest-order parameter in both cases is a second-order symmetric tensor, indicating that both species are ferroelastic. The difference, however, is that both phases of **ACA** are polar, allowing for nonzero polarization along the *c*-axis. The reduction in point symmetry determines the number of ferroelastic domains,^66^ which corresponds to the index of the low-symmetry point group within the high-symmetry group. In both cases, this number is 3. The scheme of the domains and their stress-free boundaries^40^ in this ferroic species is given in Figure S15(a). The symmetry reduction involving the loss of a threefold axis admits formation of three-arm self-similar star patterns.

**Figure 7:**
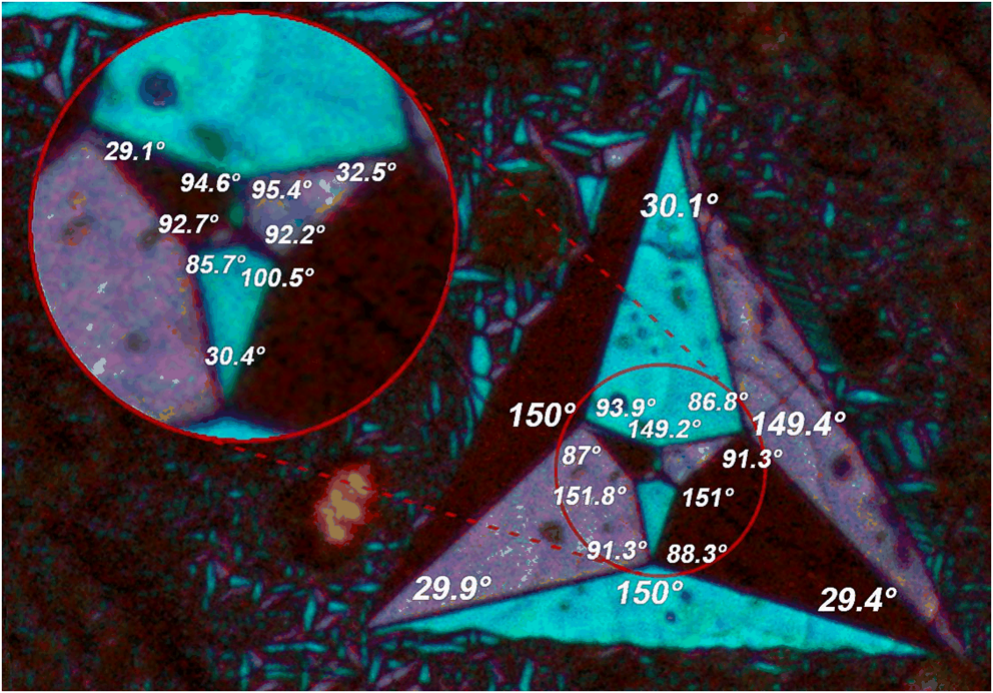
A star pattern in **ABA** at *T* = 270 K with marked selected angles.

**Figure 8:**
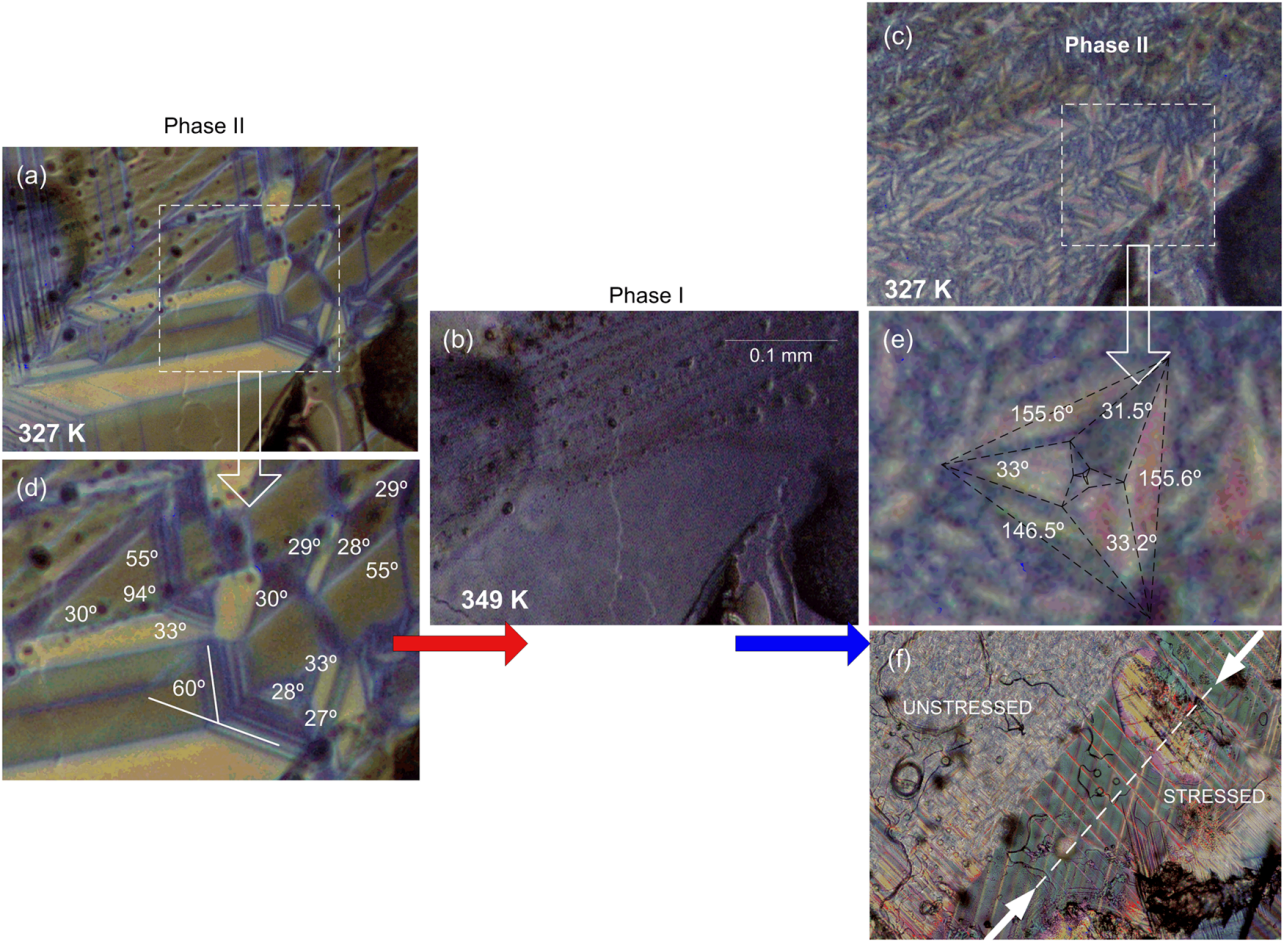
Evolution of the ferroelastic domain pattern during (a, b) heating (b, c) cooling cycles in **ACA**; (d) geometry of the ferroelastic pattern at 327 K; (e) star pattern in **ACA** at 327 K; (f) border of unstressed (upper left) and stressed (lower right) parts of **ACA** sample with the stress axis marked with a white line.

Similar star patterns have been reported until now in primarily inorganic ferroelastics showing hexagonal-to-orthorhombic symmetry reduction.^67−69^ The compounds were, however, much simpler and the content of the stars was never so abundant as here, see Figures 7, 8 (also S16 and S17). The nodes (3,1,3,5) of the boundaries schematized in Figure S15(b) are essential for the formation of star patterns because, in contrast to all other possible nodes, they involve no stresses in the adjacent domains. Therefore, the star pattern reflects the tendency of the system to minimize the elastic energy.

Details of the group-theoretical analysis of the domain pattern are given in Section S7 of SI along with numerical predictions for the angles subtended by the adjacent domain boundaries in ideal stars. The experimentally determined angles (in degrees of angle) visible in Figure 7 for **ABA**, where the star patterns are particularly abundant, are somewhat different from these predictions. The obvious reason is the presence neighboring domains that form node configurations different from the (3,1,3,5) pattern thereby introducing local stresses. The asymmetry of the nodes implied by Figure S15(a,b) in particular the difference in α_1_ and α_3_, is, however, discernible.

The ideal star pattern corresponding to the eqs (S1)–(S4) and Figure S15(a,b) is expected to appear in a defect-free sample without other domains present. It remains an intriguing technical question whether such a single star could be obtained by a specific thermal treatment or mechanical stimulation of the sample. The differences between α_1_ and α_3_, resulting from noninfinitesimal value of the in-plane spontaneous strain give rise to a vorticity in the pattern. The pattern exhibits a macroscopic circulation which in the continuous limit would give rise to a nonvanishing curl (e.g., a mirror reflection of the largest blue ray of Figure 7 in the bisector plane of the angle 30.1° interchanges the angles 86.8 and 93.9°). All the arms of this generation show the same sign of skewness. This aspect of the problem does not seem to have been discussed in the existing literature. The vorticity of the star pattern in Figure 7 is evident, with angles on the right sides of the distorted deltoidal arms consistently lower than the angles at left-hand sides, which inequality reflects the observation that α_1_ < α_3_ whenever *b*/*a* > √3.

Whereas the largest star in Figure 7 only slightly deviates from 3-fold symmetry, the nested star of the next generation shows distortion up to 2.3°. The vorticity of the outer and inner star has the same sign. Furthermore, the nested star is rotated with respect to the outer one by an average angle of −2.4° around the axis perpendicular to the plane toward the viewer, affecting all domains nominally marked with the same symbol *V*_i_, *i* = I, II, III. Further examples of star patterns are collected in SI (Section 7, Figures S16 and S17).

Figure S15 allows one to note that most stars in **ABA** are “parallel”, *i.e*. the wedges (rays of stars) of the same color being either almost parallel or antiparallel but numerous apparently perpendicular wedges are also visible in Figure S16.

Less numerous and less featured stars are also detectable in **ACA** (Figure 8(c),(e)). The observation that all angles α_2_ > 30° although  may result from stresses exerted by neighboring domains. Figure 8 demonstrates the impact of thermal history on domain pattern in **ACA**. Heating to the HT phase and subsequent cooling produces a finer domain structure, though some features remain discernible in the pristine sample. In particular, Figure 8(e) shows a star pattern alongside quasi-30° systems forming two-arm stars and plaques with shapes delineated by angles fairly close to those following from Figure S15(a). The traces of the W domain walls are consistently bright and those of the walls W′ dark, resembling the distinction between W and W′ walls based on their inclination to the observation plane.^70^ A video illustrating this evolution is provided in the SI (Movies 1 and 2).

The domain structure in **ACA** exhibits sensitivity to external stress. A qualitative investigation into this issue is illustrated in Figure 8(f). Compressive stress was applied using tweezers along the axis marked with a white line in the lower-right part of the image, while the upper-left region was left free of stress. The stressed part of Figure 8(f) displays a coarser-grained pattern with well-developed straight (planar) domain walls (bright) almost perpendicular to the stress axis. Another series of planar domain walls (pinkish) subtends an angle of about 60° with the former series. An insight into the scheme in Figure S15(a) suggests that we deal here with only one type of domain wall, likely W, while nodes involving both W and W′ have been eliminated by the stress. The stress appears to favor one of the three ferroelastic variants *V*_I_, *V*_II_ and *V*_III_ occupying the blueish regions. A schematic of a typical node in the stressed part of Figure 8(f) is provided in Figure S18, with the prevailing variant selected as *V*_I_. The actual orientation of lattice constants *a* and *b* with respect to the stress axis can only be determined with in situ crystallography. The present spatial resolution is insufficient to detail the nature of the junction between narrow regions, which, according to the present model, should be occupied by variants *V*_II_ and *V*_III_.

## Conclusions

In summary, a new group of lead-free antimony-based OIHs: (CH_3_C(NH_2_)_2_)_3_[Sb_2_Cl_9_] (**ACA**), (CH_3_C(NH_2_)_2_)_3_[Sb_2_Br_9_] (**ABA**), and (CH_3_C(NH_2_)_2_)_3_[Sb_2_I_9_] (**AIA**) has been obtained and characterized. Despite their analogous chemical compositions (R_3_Sb_2_X_9_), these materials exhibit distinct ionic arrangements in their solid phases, resulting in diverse physicochemical properties. The anionic frameworks of **ACA** and **AIA** are reminiscent of well-established ferroelectric materials, with **ACA** distinguished by its piezoelectric and ferroelastic properties, which are underpinned by a unique buckled honeycomb structure forming two-dimensional layers of antimony chloride. In contrast, **AIA** demonstrates ferroelectric attributes with a polarization of *P*_s_ = 0.2 μC cm^–1^, with discrete bioctahedral units forming a zero-dimensional structure. Intriguingly, **ABA** exhibits a surprising structural deviation from its counterparts, amalgamating the anionic subnetworks of both **ACA** and **AIA**. This results in an unprecedented hybrid two-component (0D + 2D) anionic architecture, representing a novel structural paradigm in the field of OIHs. Despite their structural differences, all three compounds share a common feature in their highest-temperature phases, which invariably exhibit a 6-fold axis of symmetry. This symmetry is subsequently reduced during PTs. The low-symmetry phases of **ACA** and **ABA** are confirmed to be orthorhombic, with one axis remaining parallel to the 6-fold axis of the HT phase. **AIA**, while following a similar scheme, presents an exception presumably due to an apparent significant multiplication of the lattice constant perpendicular to the hexagonal plane. This anomaly may be attributed to the presence of a number of stacking faults within the layers of constituent bioctahedra. Therefore, the LT phase of **AIA** has been ascribed to a general triclinic structure. The PTs hexagonal-to-orthorhombic and hexagonal-to-monoclinic are known to produce star domain patterns. To the best of our knowledge, such an abundance of these patterns in **ABA** is unprecedented in the literature, marking a significant observation in the field of domain formation in OIHs. All three compounds exhibit open band gaps, with experimentally determined absorption edges spanning the visible spectral range and confirming the presence of low-dimensional phases in these materials.

## Supporting Information Available

Crystal structure refinement data, experimental and calculated powder X-ray diffraction patterns, thermal (DSC/TGA) and SHG analysis; dielectric measurements and solid state calculations; Group-theory based predictions of the domain textures and selected results of optical observations along with the recordings illustrating evolution of the ferroelastic domain structure during heating and cooling cycles (PDF)
Traces of the W domain walls (MP4)
Traces of the W′ domain walls (MP4)
