# Supplementary material for: Ferroelectricity, Piezoelectricity, and Unprecedented Starry Ferroelastic Patterns in Organic–Inorganic (CH3C(NH2)2)3[Sb2X9] (X = Cl/Br/I) Hybrids
Source: Inorg Chem. 2025 May 5;64(19):9639–51. doi: 10.1021/acs.inorgchem.5c00667 (PMC12093296; doi:10.1021/acs.inorgchem.5c00667)
Supplement: Supplementary file 1 — ic5c00667_si_001.pdf [file ic5c00667_si_001.pdf]

Supporting Information

Ferroelectricity, Piezoelectricity, and Unprecedented Starry Ferroelastic Patterns in Organic-Inorganic $(CH_3C(NH_2)_2)_3[Sb_2X_9]$ (X=Cl/Br/I) hybrids.

Aleksandra Krupińska[1], Bogumiła Burzyńska[1], Vasyl Kinzhybalo[2], Błażej Dziuk[3], Przemysław Szklarz[1], Dariusz Kajewski[4], Jan K. Zaręba[3], Ada Drwęcka[5], Szymon J. Zelewski[5], Piotr Durlak[1], Piotr Zieliński[6], Paweł Sobieszczyk[6], Ryszard Jakubas[1] and Anna Piecha-Bisiorek[1*]

[1]Faculty of Chemistry, University of Wrocław, F. Joliot-Curie 14, 50-383 Wrocław, Poland.
[2]Institute of Low Temperature and Structure Research, Polish Academy of Science, Okólna 2, 50-422 Wrocław, Poland.
[3] Institute of Advanced Materials, Faculty of Chemistry, Wrocław University of Science and Technology, Wybrzeże Wyspiańskiego 27, 50-370 Wrocław, Poland.
[4]Institute of Physics, University of Silesia in Katowice, ul. 75 Pułku Piechoty 1, PL-41500 Chorzów, Poland.
[5]Department of Experimental Physics, Faculty of Fundamental Problems of Technology, Wrocław University of Science and Technology, 50-370 Wrocław, Poland
[6]The H. Niewodniczański Institute of Nuclear Physics PAS, Radzikowskiego 152, Kraków 31-342, Poland.

Corresponding author: Anna Piecha-Bisiorek (anna.piecha-bisiorek@uwr.edu.pl)

Table of contents:

Section 1: Experimental 2
1.1 Synthesis 2
Section 2: Thermal properties 5
Section 3: X-ray structure analysis 7
Section 4: SHG analysis 20
Section 5: Dielectric measurements 21
Section 6: Solid state static calculations 22

Section 7: Group theoretical description of domain patterns. 26
References 30

Section 1: Experimental

1.1 Synthesis

General information:

All reagents used for the syntheses of $(CH_3C(NH_2)_2)_3[Sb_2Cl_9]$ (**ACA**), $(CH_3C(NH_2)_2)_3[Sb_2Br_9]$ (**ABA**), $(CH_3C(NH_2)_2)_3[Sb_2I_9]$ (**AIA**) and were purchased from commercial sources (Sigma-Aldrich and TCI) and used without further purification: acetamidine hydrochloride ($CH_3C(=NH)NH_2{\cdot}HCl$, 97% ThermoFischer Scientific), acetamidine hydrobromide ($CH_3C(=NH)NH_2{\cdot}HBr$, >98%), acetamidinium iodide ($CH_3C(=NH)NH_2{\cdot}HI$, 98% Ossila) Sb_2O_3 (99%), SbI_3 (98%), HBr (48%), HCl (37%) and HI (57 wt. % in H_2O, distilled, stabilized, 99.95%).

Synthesis of **ACA**:

Concentrated HCl was added dropwise to a solution containing acetamidine hydrochloride and Sb_2O_3 in a molar ratio 3:1 in H_2O at 45°C. After a few days, single crystals of **ACA** were grown by slow evaporation from the colorless solution. The crystalline product was twice recrystallized from water with a small amount of HCl. As a result a transparent, a plate-like hexagonal single crystals were obtained (Figure. S1(a)).

Synthesis of **ABA**:

An aqueous solution of acetamidine hydrobromide was added to a stoichiometric amount of Sb_2O_3 (molar ratio 3:1) in HBr. The obtained precipitate was recrystallized twice from an acetonitrile solution. Yellow-transparent pseudo-hexagonal single crystals suitable for single-crystal X-ray and dielectric measurements were grown from a saturated solution by slow evaporation of an acetonitrile solution at 20°C (Figure. S1(b)).

Synthesis of **AIA**:

The crystals of **AIA** were synthesized by the slow evaporation of stochiometric amounts of acetamidine hydroiodide and antimony(III) iodide (molar ratio 3:2) with a small amount of hydroiodic acid in a methanol solution. The polycrystalline material obtained was recrystallized twice from a methanol solution (Figure. S1(c)).

The chemical composition of all compounds was confirmed by the elemental analysis (Table S1) while phase purity was verified by powder X-ray diffraction (Figure S2).

Table S1. The results of an elemental analysis for **ACA**, **ABA** and **AIA**.

**ACA**			
**Quantitative composition**	**%H**	**%C**	**%N**
Theoretical calculations	2.73	8.89	10.86
Experimental results	2.86	8.92	10.96
**ABA**			
**Quantitative composition**	**%H**	**%C**	**%N**
Theoretical calculations	2.12	6.31	7.35
Experimental results	2.15	6.34	7.38
**AIA**			
**Quantitative composition**	**%H**	**%C**	**%N**
Theoretical calculations	1.35	4.61	5.38
Experimental results	1.49	4.81	5.40

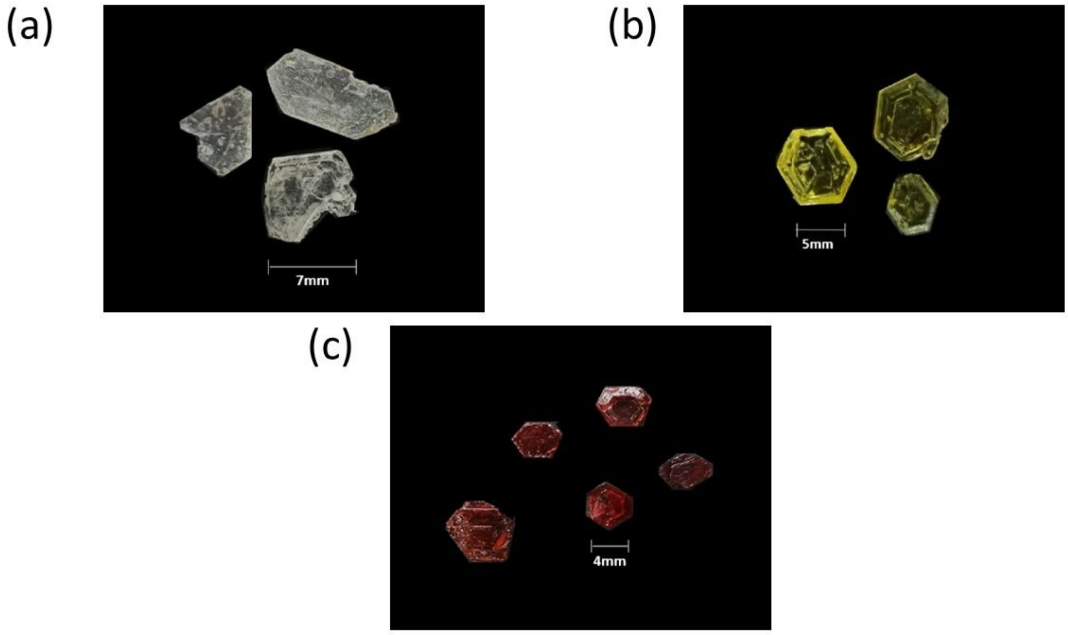

Figure S1. The photos of single-crystals of the (a) **ACA**; (b) **ABA**; (c) **AIA**.

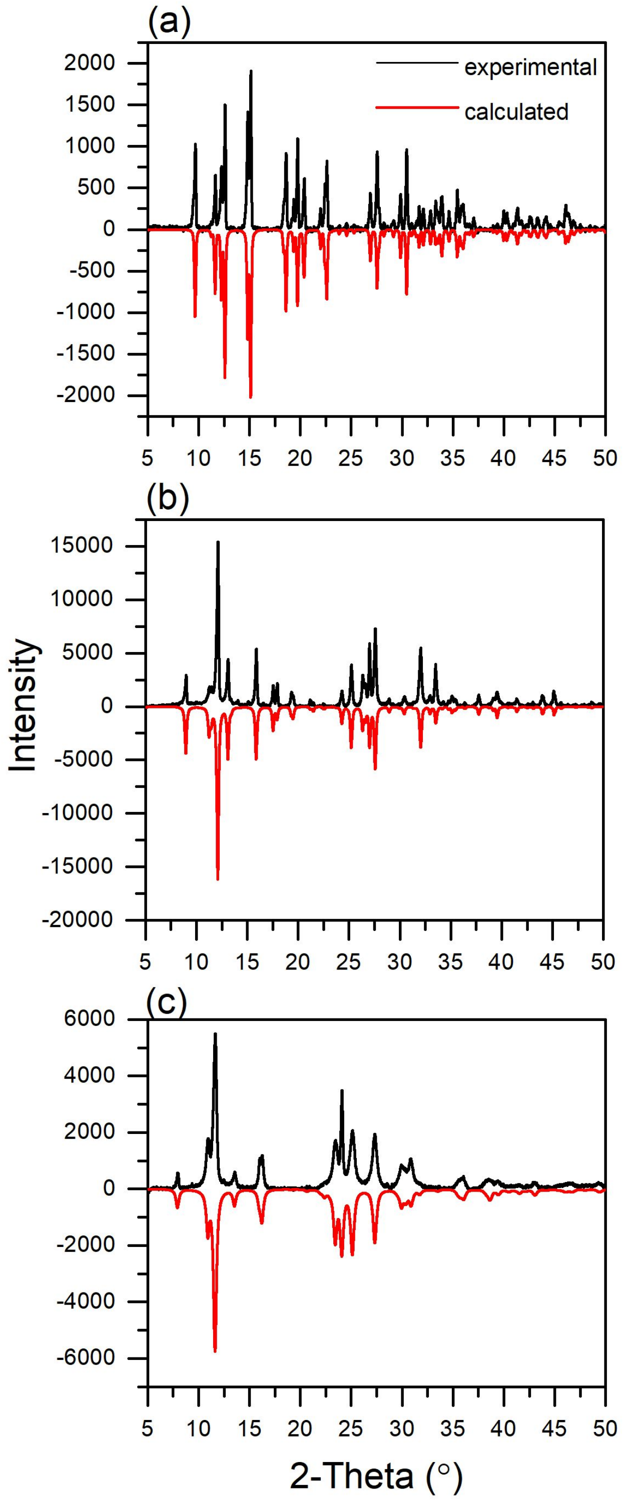

Figure S2. PXRD for (a) **ACA**, (b) **ABA** and (c) **AIA**.

Section 2: Thermal properties

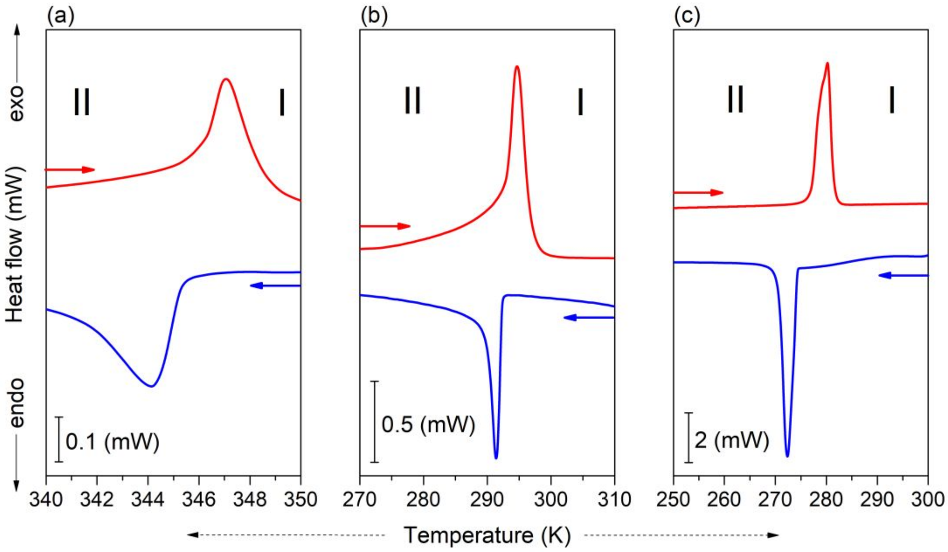

Figure S3. DSC runs measured for (a) **ACA** (m=7.63 mg), (b) **ABA (**m=17.35 mg), and (c) **AIA** (m=11.76 mg) upon heating (red) and cooling (blue) scan.

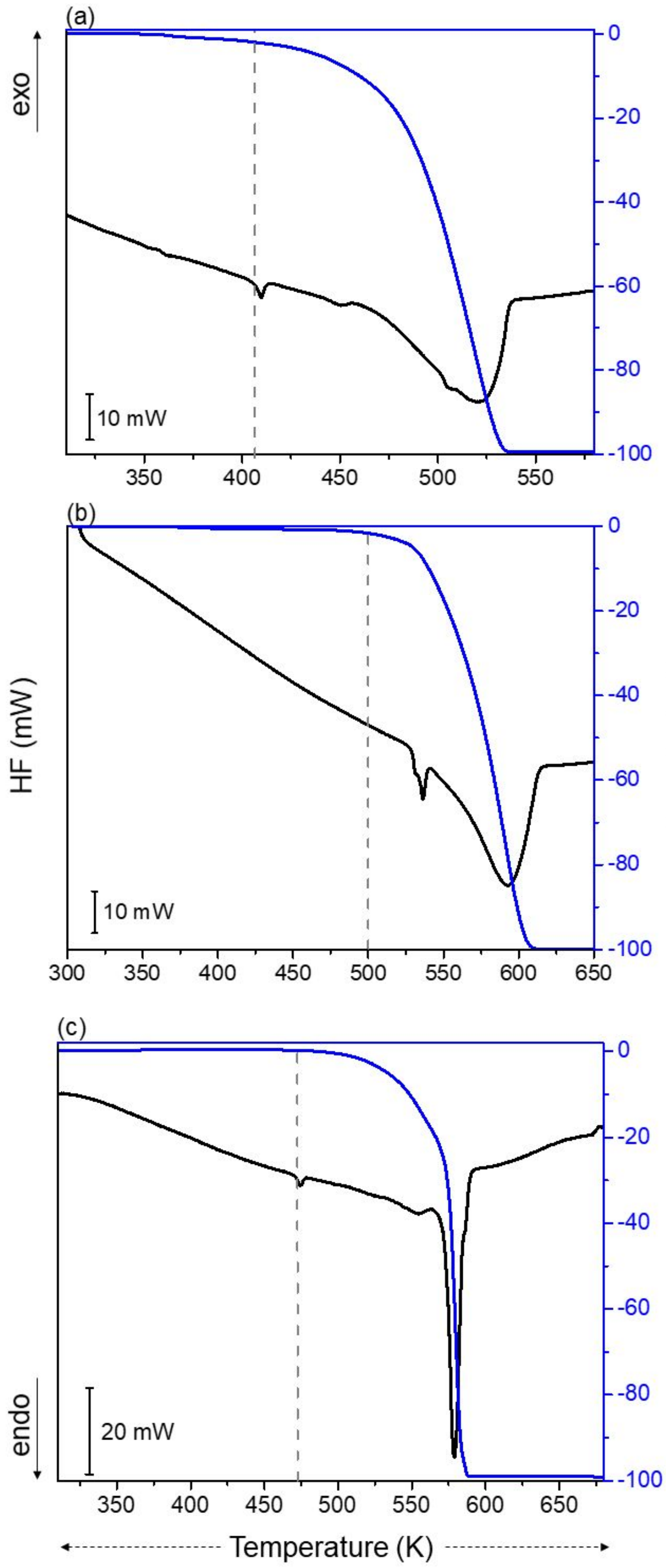

Figure S4. The results of the TGA-DTA analyses for (a) **ACA** (m=14.79 mg), (b) **ABA** (m=19.72 mg) and (c) **AIA** (m=10.53 mg).

Section 3: X-ray structure analysis

The single X-ray structure of **ACA** has been determined at 100, 293 and 382 K. The structure belongs to the orthorhombic system, space group $Cmc2_1$, at 100 K and 293 K becoming to the hexagonal system, space group $P6_3mc$, at high temperature 363 K. Table 1 contains the experimental data of single-crystal structure determination. Independent parts of LT, RT and HT phase's structures are depicted in Figure S5. The selected bond lengths and angles, as well as the geometry parameters of hydrogen bonds, are presented in Tables S2-S5.

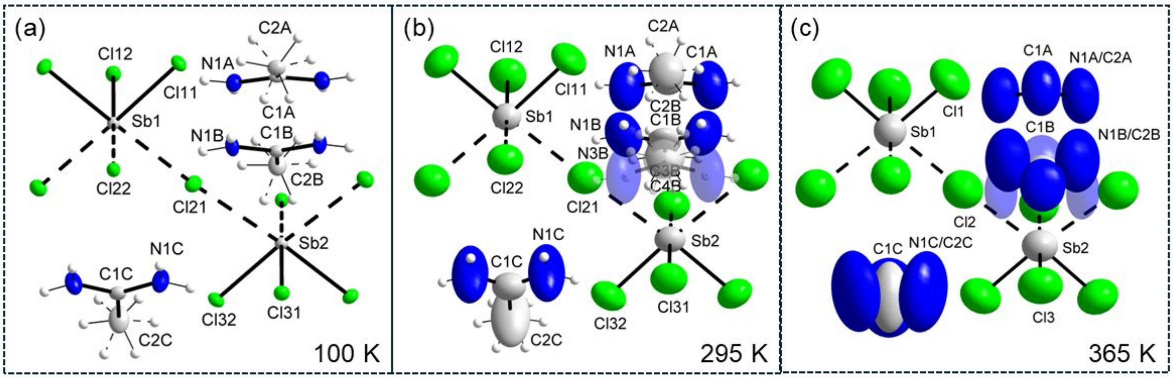

Figure S5. Independent parts of the unit cell of **ACA** at (a) 100 K (phase II); (b) 295 K (phase II); (c) 365 K (phase I) are labelled. Thermal ellipsoids are drawn at 50% probability level. Elongated Sb–Cl bonds are shown with bold broken lines. Disordered methyl groups are shown with thin broken lines. The second part of the disordered B cation is shown in transparent mode in both 295 K (b) and 365 K (c) phases.

At all temperatures the structures are composed of useful in organic–inorganic hybrids two $[SbCl_6]^{3-}$ octahedra and three acetamidinium cations (A, B and C), attracted to each other by N–H...Cl hydrogen bonds (Figure S5). At 100 K (phase (II)), all three acetamidinium cations are ordered with 50% occupation factors for atoms C1 and C2 (atom in special position lie on mirror plane). At 295 K (phase (II)), the geometrical parameters of the one of the three observed acetamidinium cations are unreasonable due to the structural character of the disorder. In this cation molecule atoms N3/N1, C3/C1 and C3/C4 are in special position and split between two positions with 25% occupancy factors. After increasing the temperature (phase (III), 363 K), release occurs of the rotation in all three cation molecules takes place. In phase (I) and (II) the octahedron atoms Cl11, Cl21 and Cl32 lies in the special position with 50% occupation factors. The distortion of the octahedra geometry is notable, with Sb–Cl distances range from 2.428 (3) to 3.055 (3) whereas the Cl–Sb–Cl angles are in the ranges

84.23 (3)– 98.81 (3) and 172.95 (4)– 177.66 (9)° (Table S3). Every chlorine atom in all temperatures is involved in the formation of hydrogen bonds as an acceptor.

Crystal packing viewed along *c*-axis direction consists of corrugated layer of two octahedral $[SbCl_6]^{3-}$ anions. Acetamidinium cations are arranged terminally and equatorially between the layers. On I → II transition the symmetry of the crystal decreases from hexagonal to orthorhombic. There is a supergroup-subgroup relation between HT and RT space groups, $P6_3mc$ and $Cmc2_1$. The volume of the orthorhombic cell is around twice of the volume of hexagonal cell. The structural changes are mainly concerned with ordering of cations. All of them are disordered in HT and become ordered, except for cation B in RT, which is disordered in two equally-occupied positions around mirror planes. Additionally, at LT all cations are ordered.

The independent part of LT structure consists of two $[SbCl_6]^{3-}$ Sb1 and Sb2 octahedra (each lying on mirror plane) and three acetamidinium cations (A, B and C), all lying on mirror planes parallel to *b* axis direction. Both carbon atoms (C1 and C2) of all cations lie on the mirror planes. The overall composition of independent part (taking into account the multiplicity of Wycoff positions) is as follows: cations A, B and C – 1/2, both Sb atoms – 1/2, Cl11, Cl21, Cl32 – 1, Cl12, Cl22 and Cl31 – 1/2; in total: $(CH_3C(NH_2)_2)_{(3/2)}Sb_{(1)}Cl_{(9/2)}$. Environment of Sb cations remains distorted octahedral with short terminal (2.4311(12)-2.4667(12) Å) and long bridging (2.8545(10)-2.9958(11)) Sb–Cl distances.

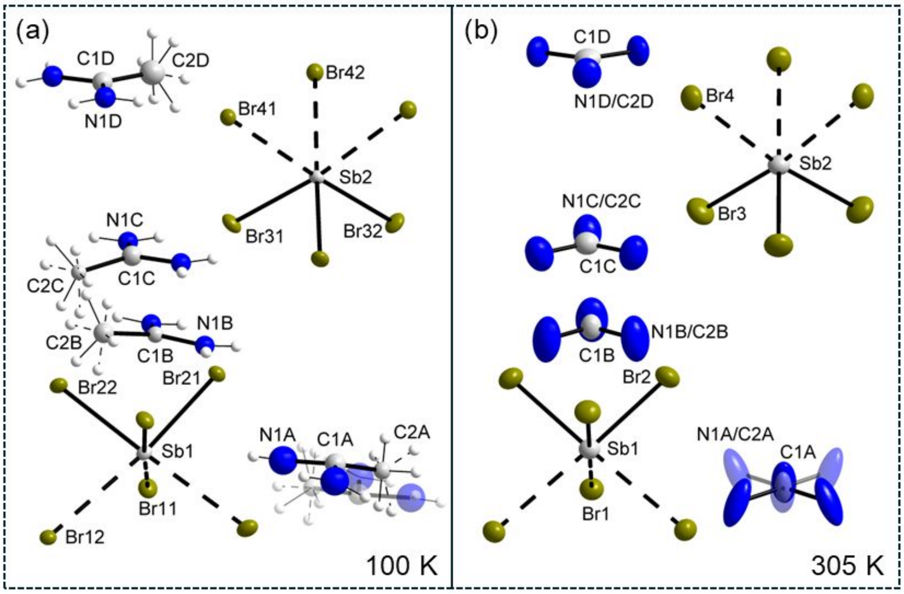

Figure S6. Independent parts of LT (a) and HT (b) phase structures of **ABA** are labeled. Thermal ellipsoids are drawn at 10% (a) and 50% (b) probability level. Elongated Sb–Br bonds are shown with bold broken lines. Disordered methyl groups are shown with thin broken lines. The second part of the disordered A cation in LT phase (a) is shown in transparent mode.

The **ABA** compound crystallizes in hexagonal space group type ($P6_3/mmc$) at RT. The independent part of the crystal structure (Figure S6) consists of two $[SbBr_6]^{3-}$ Sb1 and Sb2 octahedra (each lying on six-fold type axes) and four acetamidinium cations (A, B, C and D), all disordered around six fold-type axes, exhibiting free rotation in *ab* plane (Figure S6(a)). Central carbon atoms (C1) lie on six-fold axes, whereas terminal NH_2 (N1) and CH_3 (C2) groups occupy common positions with 2/3 and 1/3 occupancy factors, respectively. The overall composition of independent part (taking into account the multiplicity of Wycoff positions) is as follows: cations A and D – 1/12, cations B and C – 1/6, both Sb atoms – 1/6, Br1 and Br4 – 1/12, Br2 and Br3 – 1/6; in total: $(CH_3C(NH_2)_2)_{(1/2)}Sb_{(1/3)}Br_{(3/2)}$. Both Sb cations possess distorted octahedral environment of Br anions. Terminal bonds (Sb1–Br2, 2.614(2) Å and Sb2–Br3, 2.611(3) Å) are much shorter than bridging ones (Sb1–Br1, 3.1231(14) Å and Sb2–Br4, 3.085(3) Å). Bridging Br1 ions produce infinite 2D hexagonal net built of Sb1 vertex-sharing octahedra. Whereas bridging Br4 ions give 0D face-sharing Sb2 bioctaedra (Table S6).

Phase transition from phase I to phase II takes place at 292 K and results in the appearance of domain structure (Figure 7) as evidenced by optical microscopy studies. Single-crystal X-ray diffraction studies indicate extensive twinning of the crystal in phase II, with 6 types of domains rotated by ~60°, ~120° and ~180° in respect to each other around the direction of 6-fold axis of phase I (Figure S7). On I → II transition the symmetry of the crystal decreases from hexagonal to orthorhombic. There is a supergroup-subgroup relation between HT and LT space groups, $P6_3/mmc$ and *Cmcm*. The volume of the orthorhombic cell is around twice of the volume of hexagonal cell.

The structural changes are mainly concerned with ordering of cations. All of them become ordered, except for cation A, which is disordered in two equally-occupied positions around $2/m$ point at (1/2, 1/2, 0). The numbering scheme and independent part selection in the LTP was done so it is consistent with HTP. The independent part of LTP structure consists of two $[SbBr_6]^{3-}$ Sb1 and Sb2 octahedra (each lying on mirror plane) and four acetamidinium cations (A, B, C and D), all lying on mirror planes perpendicular to *a*-axis direction (Figure. S6(b)). Both carbon atoms (C1 and C2) of all cations lie on the mirror planes, additionally the D cation occupies *m*2*m* site, with 2-fold axis going along C1D–C2D bond. The overall composition of independent part (taking into account the multiplicity of Wycoff positions) is as follows: cations A and D – 1/4, cations B and C – 1/2, both Sb atoms – 1/2, Br11, Br21, Br32 and Br42 – 1/2, Br12 and Br41 – 1/4, Br22 and Br31 – 1; in total:

$(CH_3C(NH_2)_2)_{(3/2)}Sb_{(1)}Br_{(9/2)}$. Environment of Sb cations remains distorted octahedral with short terminal (2.604(5)-2.631(3) Å) and long bridging (3.045(4)-3.162(3)) Sb–Br distances.

Table S2. Selected geometric parameters (Å, °) for 100, 295 and 365 K for **ACA**.

**100 K**			
Sb1—Cl11	2.4833 (9)	Sb1—Cl22	2.9869 (14)
Sb1—Cl11^{i}	2.4833 (9)	Sb2—Cl21	2.9958 (11)
Sb1—Cl12	2.4667 (12)	Sb2—Cl31	2.4311 (12)
Sb1—Cl21^{i}	2.8545 (10)	Sb2—Cl32	2.4320 (9)
Sb1—Cl21	2.8545 (10)	Sb2—Cl32^{ii}	2.4319 (9)
Cl11^{i}—Sb1—Cl11	89.13 (4)	Cl21^{i}—Sb1—Cl22	98.80 (3)
Cl11—Sb1—Cl21	90.58 (3)	Cl31—Sb2—Cl21	86.53 (3)
Cl11^{i}—Sb1—Cl21	176.91 (3)	Cl31—Sb2—Cl32^{ii}	90.85 (3)
Cl11^{i}—Sb1—Cl21^{i}	90.58 (3)	Cl32—Sb2—Cl21	86.20 (3)
Cl11—Sb1—Cl22	84.23 (3)	Cl32^{ii}—Sb2—Cl21	176.41 (3)
Cl12—Sb1—Cl11^{i}	90.76 (3)	Cl32^{ii}—Sb2—Cl32	91.39 (4)
Cl12—Sb1—Cl21^{i}	86.17 (3)	Sb1—Cl21—Sb2	176.69 (3)
Cl12—Sb1—Cl22	172.95 (4)	Sb1—Cl21—Sb2^{ii}	176.69 (3)
Cl21—Sb1—Cl21^{i}	89.54 (4)		
**295 K**			
Sb1—Cl11^{i}	2.480 (2)	Sb1—Cl22	3.044 (4)
Sb1—Cl11	2.480 (2)	Sb2—Cl21	3.055 (3)
Sb1—Cl12	2.434 (3)	Sb2—Cl31	2.428 (3)
Sb1—Cl21^{i}	2.904 (3)	Sb2—Cl32	2.434 (2)
Sb1—Cl21	2.904 (3)	Sb2—Cl32^{ii}	2.434 (2)
Cl11—Sb1—Cl11^{i}	89.95 (12)	Cl21^{i}—Sb1—Cl22	95.56 (8)
Cl11—Sb1—Cl21	88.49 (8)	Cl31—Sb2—Cl21	86.17 (7)
Cl11^{i}—Sb1—Cl21	177.65 (9)	Cl31—Sb2—Cl32^{ii}	90.47 (8)
Cl11^{i}—Sb1—Cl21^{i}	88.49 (8)	Cl32—Sb2—Cl21	86.38 (8)
Cl11^{i}—Sb1—Cl22	86.08 (8)	Cl32^{ii}—Sb2—Cl21	175.67 (8)
Cl12—Sb1—Cl11^{i}	90.47 (9)	Cl32^{ii}—Sb2—Cl32	90.93 (12)
Cl12—Sb1—Cl21	87.80 (9)	Sb1—Cl21—Sb2	178.21 (11)
Cl12—Sb1—Cl22	175.11 (13)	Sb2^{iii}—Cl22—Sb1	175.37 (16)
Cl21^{i}—Sb1—Cl21	93.02 (11)		
**365 K**			
Sb1—Cl1^{iv}	2.461 (5)	Sb2—Cl2	3.052 (9)
Sb1—Cl2	2.977 (8)	Sb2—Cl3^{v}	2.434 (5)
Cl1^{iv}—Sb1—Cl1	90.3 (2)	Cl3^{v}—Sb2—Cl2	175.0 (2)
Cl1—Sb1—Cl2	87.85 (13)	Cl3^{v}—Sb2—Cl3^{vi}	90.70 (19)
Cl1^{iv}—Sb1—Cl2	177.4 (2)	Sb1—Cl2—Sb2	177.3 (3)
Cl3^{vi}—Sb2—Cl2	85.78 (11)		

Symmetry code(s): (i) -*x*+1, *y*, *z*; (ii) -*x*+2, *y*, *z*; (iii) *x*-1/2, *y*-1/2, *z*; (iv) -*y*+1, *x*-*y*, *z*; (v) -*x*+*y*+1, -*x*+2, *z*; (vi) -*y*+2, *x*-*y*+1, *z*.

Table S3. Hydrogen-bond geometry (Å, º) for **ACA** at 100 K.

D—H···*A*	*D*—H	H···*A*	*D*···*A*	*D*—H···*A*
N1*A*—H1*AA*···Cl11^{i}	0.88	2.51	3.369 (3)	166
N1*A*—H1*AB*···Cl12	0.88	2.64	3.521 (3)	177
C2*A*—H2*AA*···Cl21	0.98	2.87	3.723 (4)	146
C2*A*—H2*AB*···Cl32^{ii}	0.98	2.91	3.679 (4)	136
C2*A*—H2*AC*···Cl11^{iii}	0.98	2.79	3.608 (3)	141
N1*B*—H1*BA*···Cl21^{iv}	0.88	2.43	3.231 (3)	151
N1*B*—H1*BB*···Cl22	0.88	2.44	3.287 (3)	163
C2*B*—H2*BB*···Cl21^{iii}	0.98	2.84	3.617 (4)	137
C2*B*—H2*BC*···Cl11	0.98	2.84	3.737 (4)	153
N1*C*—H1*CA*···Cl22	0.88	2.84	3.436 (3)	127
N1*C*—H1*CA*···Cl32^{iv}	0.88	2.64	3.429 (3)	150
N1*C*—H1*CB*···Cl31	0.88	2.67	3.541 (3)	171
C2*C*—H2*CB*···Cl32	0.98	2.89	3.738 (4)	145
C2*C*—H2*CC*···Cl32^{v}	0.98	2.94	3.738 (4)	139

Symmetry codes: (i) −*x*+3/2, *y*+1/2, *z*; (ii) *x*, −*y*+1, *z*+1/2; (iii) −*x*+2, *y*, *z*; (iv) −*x*+3/2, *y*−1/2, *z*; (v) −*x*+1, *y*, *z*.

Table S4. Hydrogen-bond geometry (Å, º) for **ACA** at 295 K.

D—H···*A*	*D*—H	H···*A*	*D*···*A*	*D*—H···*A*
N1*A*—H1*AA*···Cl11^{i}	0.86	2.63	3.474 (8)	167
N1*A*—H1*AB*···Cl12	0.86	2.70	3.559 (9)	172
C2*A*—H2*AA*···Cl21	0.96	3.23	3.708 (12)	113
C2*A*—H2*AB*···Cl11^{ii}	0.96	3.10	3.591 (8)	114
N1*B*—H1*BA*···Cl21^{iii}	0.86	2.58	3.34 (2)	147
N1*B*—H1*BB*···Cl22	0.86	2.60	3.43 (2)	163
C2*B*—H2*BA*···Cl21	0.96	2.78	3.56 (2)	139
C2*B*—H2*BB*···Cl11^{ii}	0.96	2.98	3.84 (3)	149
N3*B*—H3*BA*···Cl22	0.86	2.57	3.33 (2)	148
N3*B*—H3*BB*···Cl21	0.86	2.61	3.38 (2)	149
C4*B*—H4*BA*···Cl21^{iii}	0.96	2.63	3.41 (2)	139
C4*B*—H4*BB*···Cl32^{iv}	0.96	2.85	3.62 (3)	139
C4*B*—H4*BC*···Cl21^{iv}	0.96	2.73	3.41 (2)	129
N1*C*—H1*CA*···Cl22	0.86	2.92	3.542 (12)	130
N1*C*—H1*CA*···Cl32^{iii}	0.86	2.74	3.506 (12)	149
N1*C*—H1*CB*···Cl31	0.86	2.80	3.644 (10)	167
C2*C*—H2*CA*···Cl32^{v}	0.96	3.08	3.847 (14)	138

Symmetry code(s): (i) −*x*+3/2, *y*+1/2, *z*; (ii) −*x*+2, *y*, *z*; (iii) −*x*+3/2, *y*−1/2, *z*; (iv) *x*+1/2, *y*−1/2, *z*; (v) −*x*+1, *y*, *z*.

Table S5. Hydrogen-bond geometry (Å, º) for **ACA** at 365 K.

D—H···*A*	*D*—H	H···*A*	*D*···*A*	*D*—H···*A*
N1*A*—H1*AA*···Cl1^{i}	0.86	2.67	3.518 (11)	168
N1*A*—H1*AB*···Cl1	0.86	2.67	3.518 (11)	168
C2*A*—H2*AB*···Cl2^{ii}	0.96	2.93	3.78 (2)	149
C2*A*—H2*AC*···Cl1	0.96	2.64	3.518 (11)	152
N1*B*—H1*BA*···Cl1^{i}	0.86	2.95	3.79 (5)	166
N1*B*—H1*BB*···Cl2	0.86	2.69	3.53 (4)	166
C2*B*—H2*BA*···Cl2^{ii}	0.96	2.76	3.53 (4)	138
C2*B*—H2*BB*···Cl1	0.96	2.92	3.79 (5)	151
C2*B*—H2*BC*···Cl2	0.96	2.74	3.53 (4)	140
N3*B*—H3*BA*···Cl2	0.86	2.73	3.35 (3)	130
N3*B*—H3*BA*···Cl3	0.86	2.95	3.70 (3)	147
N3*B*—H3*BB*···Cl2^{iii}	0.86	2.53	3.35 (3)	160
C4*B*—H4*BA*···Cl2	0.96	2.52	3.35 (3)	145
C4*B*—H4*BB*···Cl2^{iii}	0.96	2.51	3.35 (3)	145
N1*C*—H1*CA*···Cl3^{iv}	0.86	2.84	3.676 (17)	163
N1*C*—H1*CB*···Cl3	0.86	2.85	3.676 (17)	161
C2*C*—H2*CA*···Cl3^{iv}	0.96	2.84	3.676 (17)	147
C2*C*—H2*CC*···Cl1^{v}	0.96	2.83	3.74 (3)	160

Symmetry code(s): (i) -*y*+1, *x*-*y*+1, *z*; (ii) -*x*+*y*+1, -*x*+2, *z*; (iii) -*y*+1, *x*-*y*, *z*; (iv) -*x*+*y*, -*x*+1, *z*; (v) -*x*+1, -*y*+1, *z*+1/2.

(a)

(b)

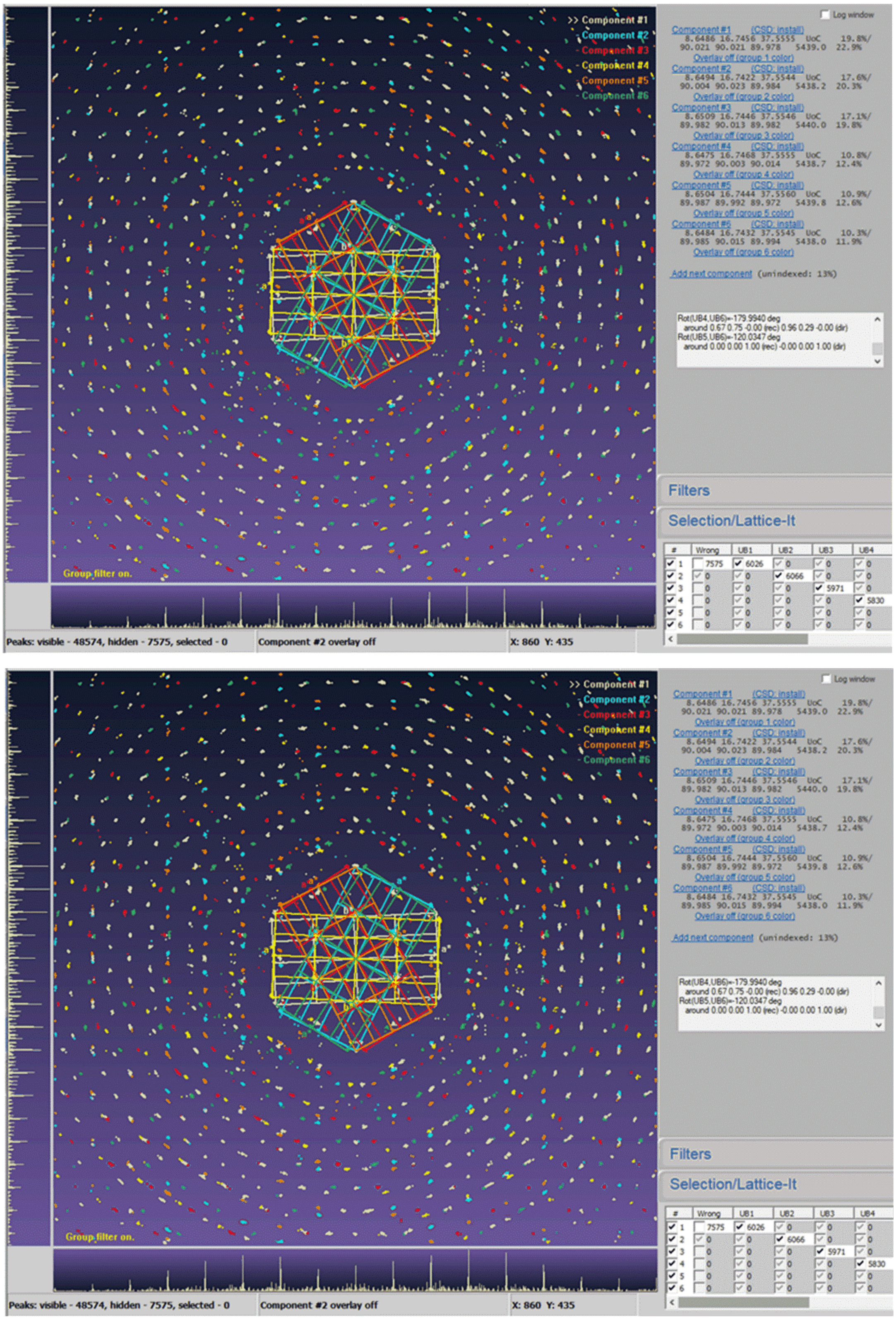

Figure S7. The view of LT phase six-component twin Ewald sphere in **ABA**. Projection along the direction (a) and perpendicular to the direction (b) of six-fold axis of HT phase.

Table S6. Selected geometric parameters (Å, °) for 100 and 305 K for **ABA**.

**100 K**		**305 K**	
Sb1—Br11	3.0555 (19)	Sb1—Br1	3.1231 (14)
Sb1—Br11^{iv}	3.0555 (19)	Sb1—Br1^{i}	3.1231 (14)
Sb1—Br12	3.162 (3)	Sb1—Br1^{ii}	3.1231 (14)
Sb1—Br21	2.604 (5)	Sb1—Br2	2.614 (2)
Sb1—Br22	2.613 (3)	Sb1—Br2^{i}	2.614 (2)
Sb1—Br22^{i}	2.613 (3)	Sb1—Br2^{ii}	2.614 (2)
Sb2—Br31	2.631 (3)	Sb2—Br3	2.611 (3)
Sb2—Br31^{i}	2.631 (3)	Sb2—Br3^{iii}	2.611 (3)
Sb2—Br32	2.615 (5)	Sb2—Br3^{iv}	2.611 (3)
Sb2—Br41	3.077 (5)	Sb2—Br4	3.085 (3)
Sb2—Br42	3.045 (4)	Sb2—Br4^{iii}	3.085 (3)
Sb2—Br42^{ii}	3.045 (4)	Sb2—Br4^{iv}	3.084 (3)
Br11—Sb1—Br12	98.30 (6)	Br1^{ii}—Sb1—Br1^{i}	94.00 (4)
		Br1^{ii}—Sb1—Br1	94.00 (4)
		Br1^{i}—Sb1—Br1	94.00 (4)
Br21—Sb1—Br11	86.52 (9)	Br2^{ii}—Sb1—Br1^{ii}	87.37 (4)
Br21—Sb1—Br12	173.15 (14)	Br2—Sb1—Br1^{i}	177.98 (8)
Br22—Sb1—Br11	89.39 (7)	Br2—Sb1—Br1	87.37 (4)
Br22—Sb1—Br12	83.95 (9)	Br2—Sb1—Br1^{ii}	87.37 (4)
Br22^{i}—Sb1—Br11	177.70 (12)	Br2^{ii}—Sb1—Br1	177.98 (8)
		Br2^{i}—Sb1—Br1^{ii}	177.98 (8)
Br22^{i}—Sb1—Br12	83.95 (9)	Br2^{i}—Sb1—Br1	87.38 (4)
		Br2^{ii}—Sb1—Br1^{i}	87.37 (4)
		Br2^{i}—Sb1—Br1^{i}	87.37 (4)
Br22^{i}—Sb1—Br22	91.31 (15)	Br2^{ii}—Sb1—Br2^{i}	91.21 (9)
Br21—Sb1—Br22	91.28 (11)	Br2—Sb1—Br2^{i}	91.21 (9)
Br21—Sb1—Br22^{i}	91.28 (11)	Br2^{ii}—Sb1—Br2	91.21 (9)
Br31—Sb2—Br31^{i}	93.37 (15)	Br3^{iii}—Sb2—Br3	93.64 (11)
Br32—Sb2—Br31	94.07 (11)	Br3^{iv}—Sb2—Br3	93.64 (11)
Br32—Sb2—Br31^{i}	94.07 (11)	Br3^{iii}—Sb2—Br3^{iv}	93.64 (11)
Br31—Sb2—Br41	87.87 (10)	Br3^{iii}—Sb2—Br4	91.42 (7)
Br31^{i}—Sb2—Br41	87.87 (10)	Br3^{iv}—Sb2—Br4^{iv}	91.42 (7)
Br31^{i}—Sb2—Br42^{ii}	91.68 (10)	Br3^{iv}—Sb2—Br4^{iii}	91.42 (7)
Br31—Sb2—Br42	91.68 (10)	Br3—Sb2—Br4^{iv}	91.42 (7)
Br31^{i}—Sb2—Br42	173.20 (12)	Br3—Sb2—Br4^{iii}	172.60 (10)
Br31—Sb2—Br42^{ii}	173.20 (12)	Br3^{iv}—Sb2—Br4	172.60 (10)
Br32—Sb2—Br41	177.16 (15)	Br3^{iii}—Sb2—Br4^{iv}	172.60 (10)
Br32—Sb2—Br42^{ii}	90.10 (11)	Br3—Sb2—Br4	91.42 (7)
Br32—Sb2—Br42	90.10 (11)	Br3^{iii}—Sb2—Br4^{iii}	91.42 (7)
Br42—Sb2—Br41	87.77 (10)	Br4^{iv}—Sb2—Br4^{iii}	83.06 (7)
Br42^{ii}—Sb2—Br41	87.77 (10)	Br4—Sb2—Br4^{iv}	83.06 (7)
Br42—Sb2—Br42^{ii}	82.94 (14)	Br4—Sb2—Br4^{iii}	83.06 (7)
Sb2^{iii}—Br42—Sb2	76.13 (13)		

Symmetry codes for 100 K: (i) −*x*+1, *y*, *z*; (ii) −*x*+1, *y*, −*z*+1/2; (iii) *x*, *y*, −*z*+1/2; (iv) *x*+1/2, –*y*+1/2, –z).
Symmetry codes for 305 K: (i) −*y*+1, *x*–*y*+1, *z*; (ii) −*x*+*y*, −*x*+1, *z*; (iii) −*y*, *x*–*y*, *z*; (iv) −*x*+*y*, −*x*, *z*.

Table S7. Hydrogen-bond geometry (Å, º) for **ABA** at 100 K.

D—H···*A*	*D*—H	H···*A*	*D*···*A*	*D*—H···*A*
N1*A*—H1*AA*···Br12^{iv}	0.88	2.54	3.39 (6)	161
N1*A*—H1*AB*···Br11	0.88	2.60	3.31 (6)	140
N1*B*—H1*BA*···Br12^{iv}	0.88	3.12	3.62 (2)	118
N1*B*—H1*BA*···Br22^{v}	0.88	2.69	3.52 (2)	158
N1*B*—H1*BB*···Br21	0.88	2.71	3.59 (2)	177
N1*C*—H1*CA*···Br31	0.88	2.63	3.49 (2)	164
N1*C*—H1*CB*···Br32^{vi}	0.88	2.73	3.60 (2)	168
N1*D*—H1*DA*···Br42^{vii}	0.88	2.52	3.36 (3)	160
N1*D*—H1*DB*···Br41	0.88	2.59	3.46 (4)	171

Symmetry codes: (iv) *x*−1/2, *y*+1/2, *z*; (v) −*x*+1/2, *y*+1/2, *z*; (vi) *x*−1/2, *y*−1/2, *z*; (vii) −*x*+1/2, *y*−1/2, −*z*+1/2.

Figure S8. Cell parameters for **AIA** along with Ewald sphere cuts for LTP and HTP.

	LTP-**AIA**	HTP-**AIA**
	Crystal system unknown<br>a = 9.4199(11) b = 9.4217(10) c = 63.329(4)<br>α = 90.052(7) β = 90.118(7) γ = 119.967(12)<br>V = 4869.1(8)	Hexagonal<br>a = 9.3283(15) b = 9.3283(15) c = 22.126(3)<br>α = 90.0 β = 90.0 γ = 120.0<br>V = 1667.4(5)
h0l	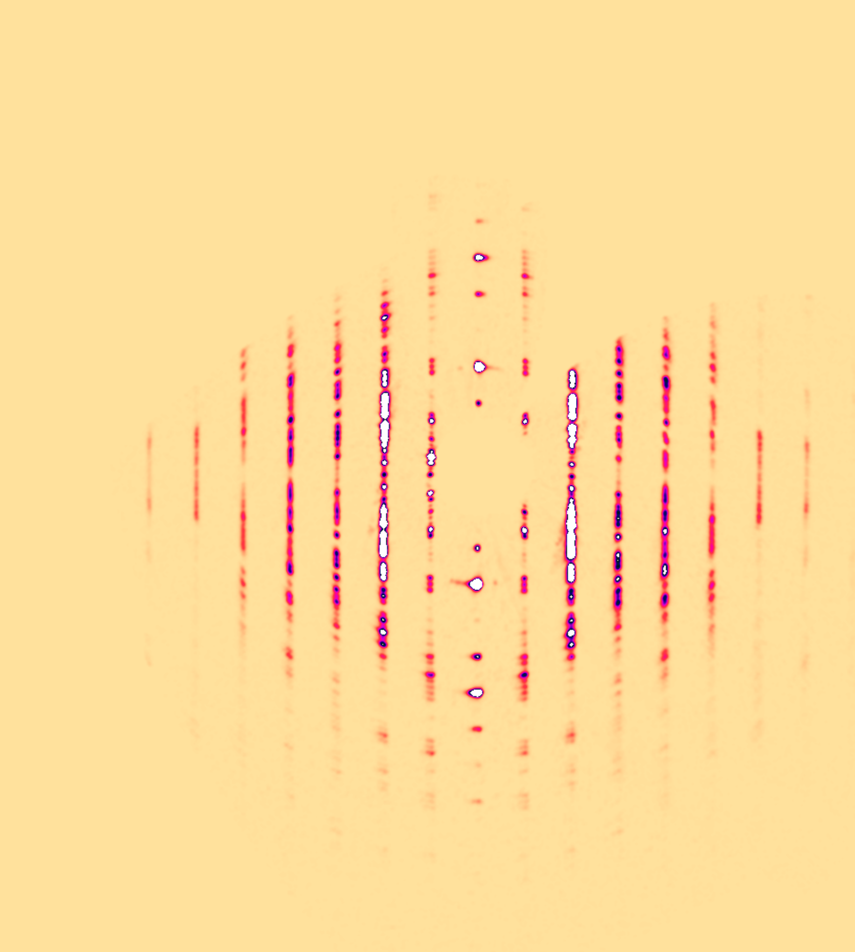	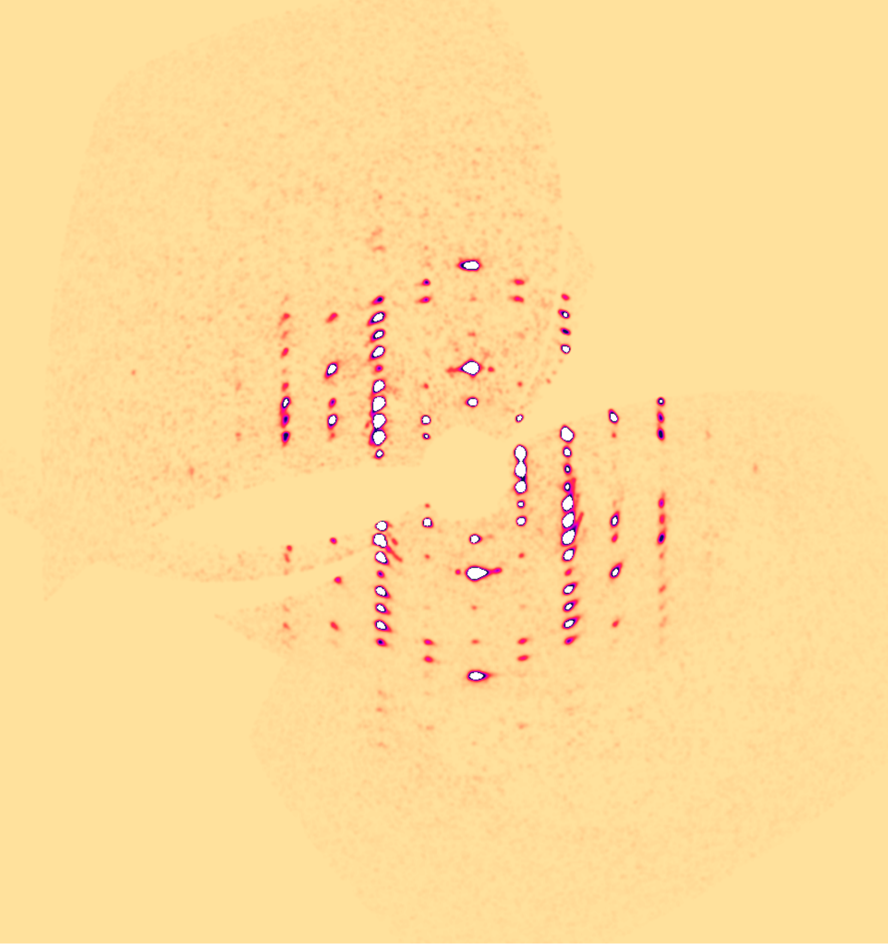

h1l

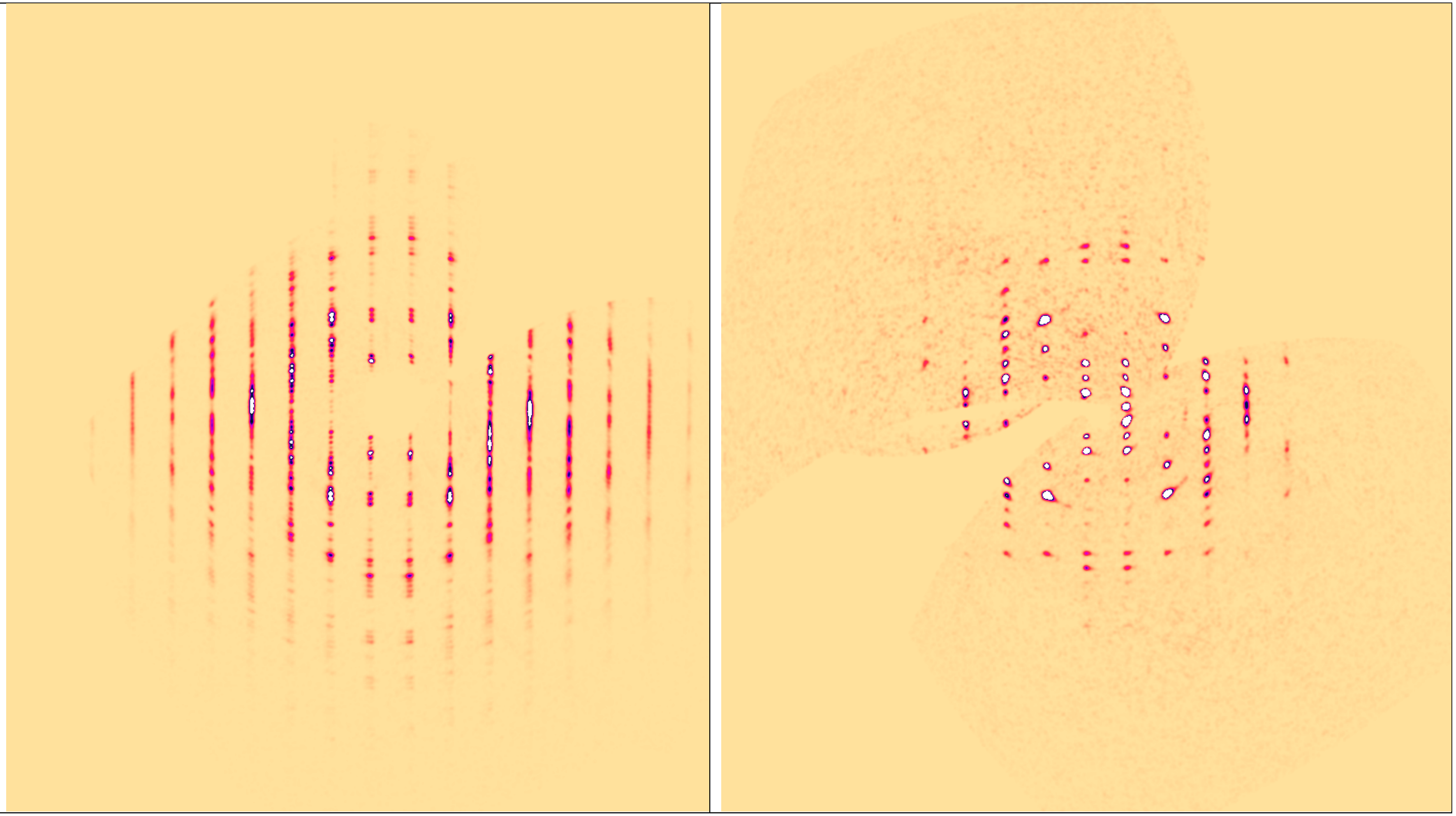

0kl

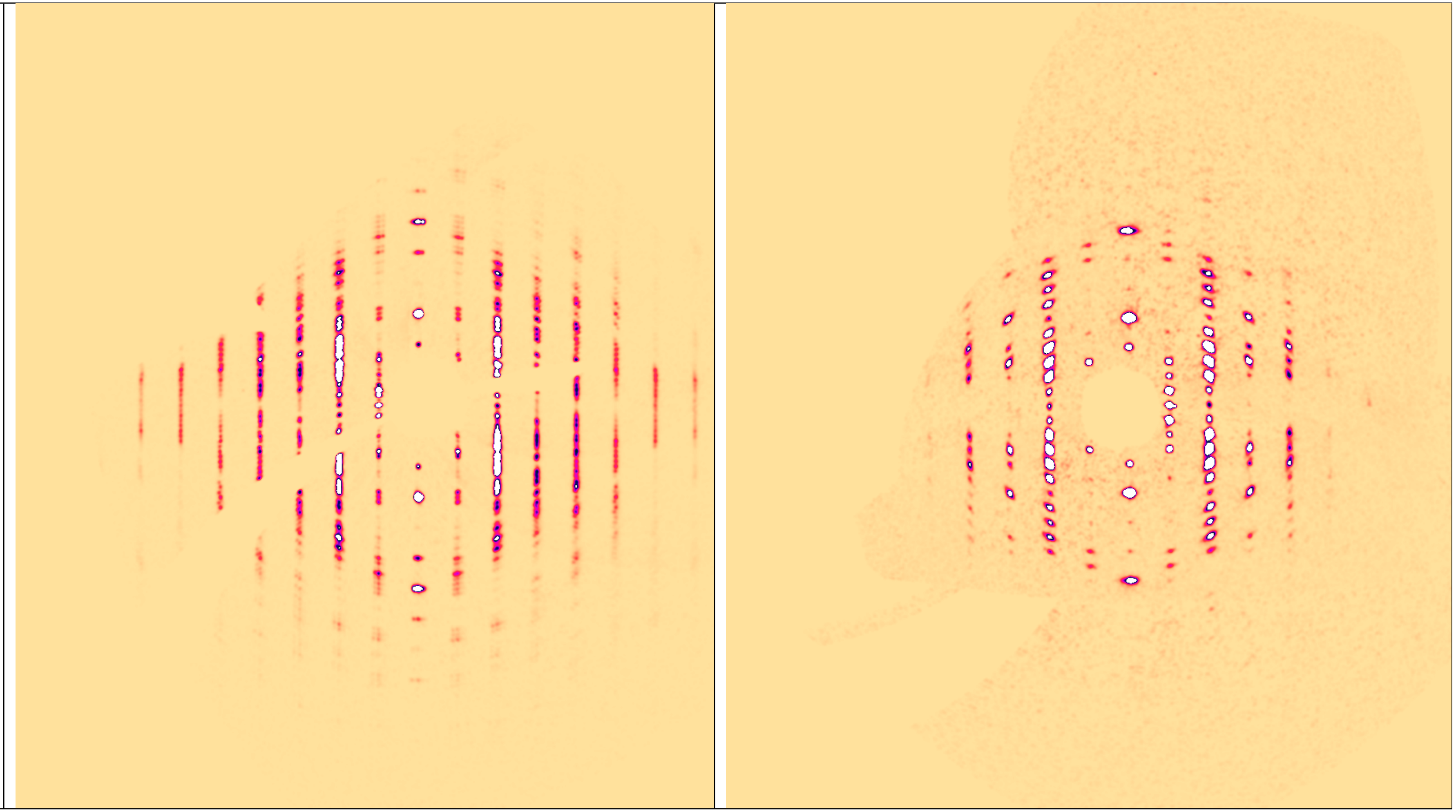

1kl

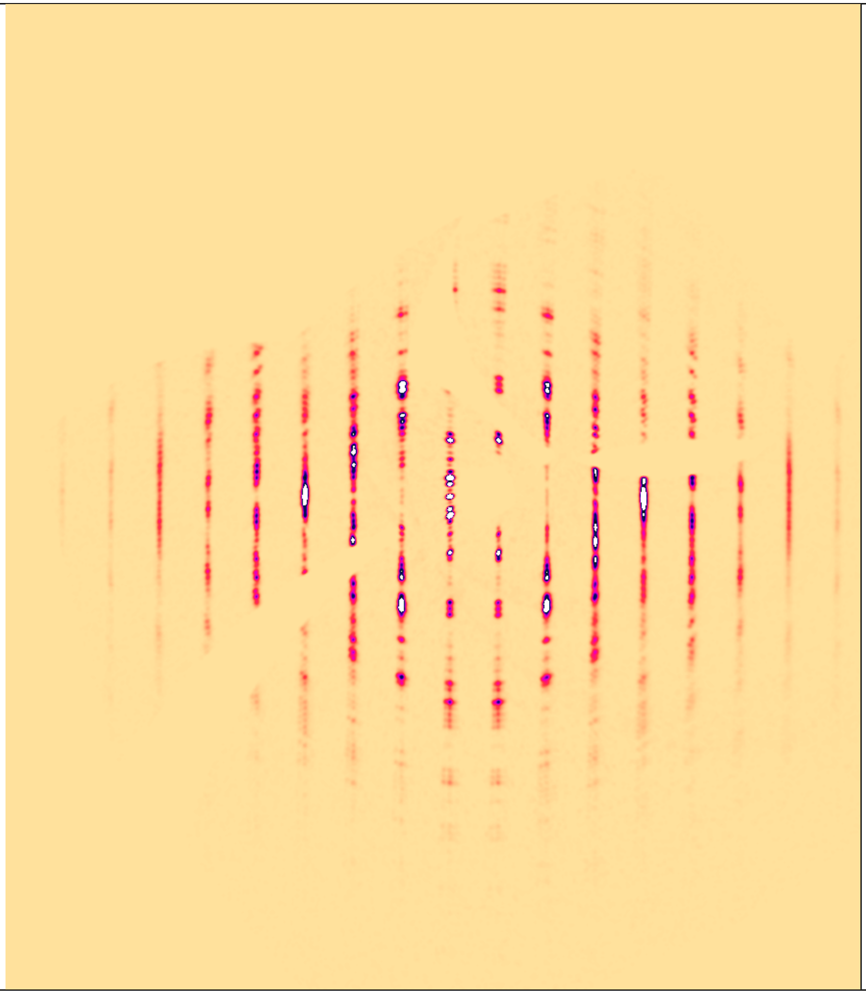

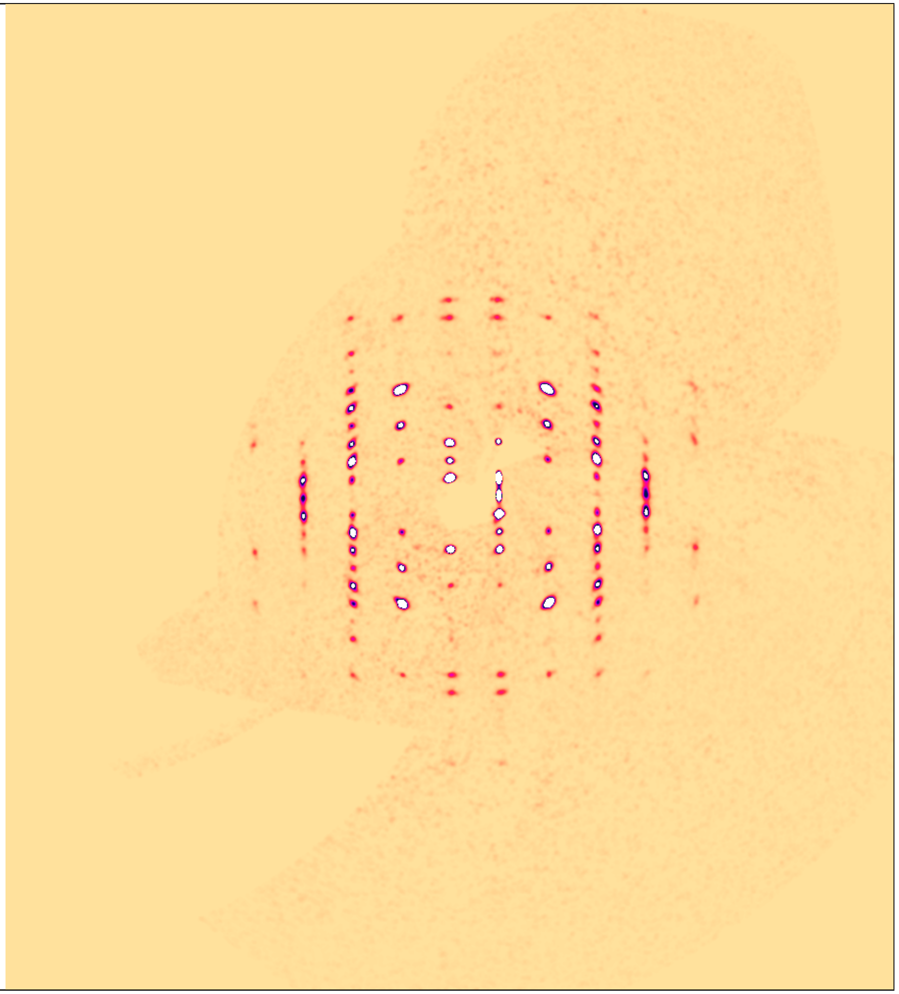

Section 4: SHG analysi

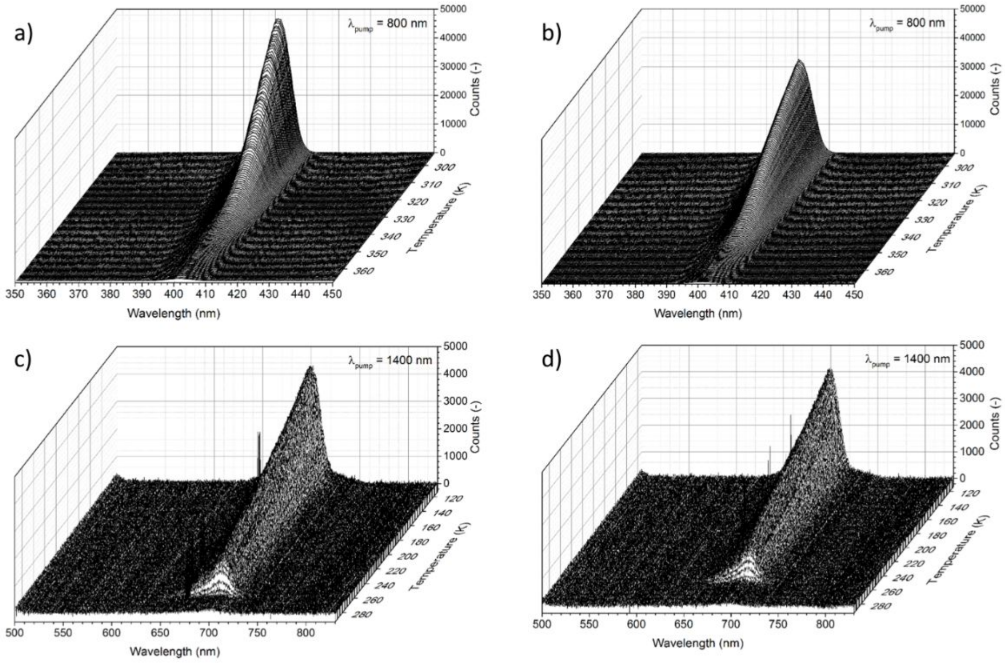

Figure S9. Overlay of experimental SHG spectra obtained upon irradiation with 800 nm femtosecond laser pulses of **ACA** for a) heating and b) cooling in 293 K – 368 K range. Overlay of experimental SHG spectra obtained upon irradiation with 1400 nm femtosecond laser pulses of **AIA** for c) heating and d) cooling in 113 K – 293 K range.

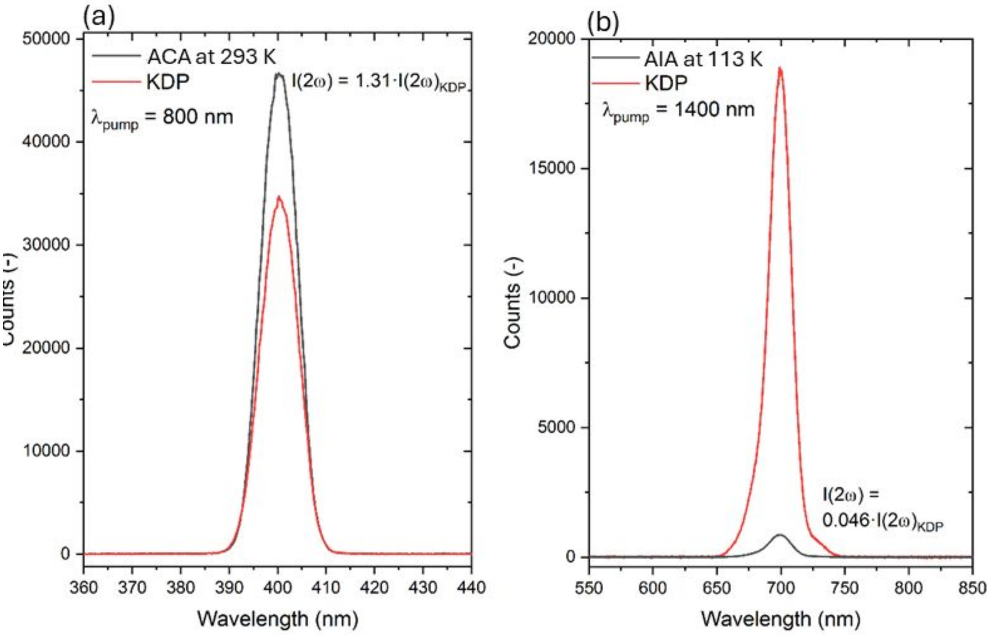

Figure S10. (a) Overlay of SHG trace of **ACA** with that of KDP obtained upon irradiation with 800 nm femtosecond laser pulses at 293 K. (b) Overlay of SHG trace of **AIA** with that of KDP obtained upon irradiation with 1400 nm femtosecond laser pulses. Signals for **AIA** and KDP were obtained at 113 K and 293 K, respectively. SHG traces are normalized to the same integration time.

Section 5: Dielectric measurements

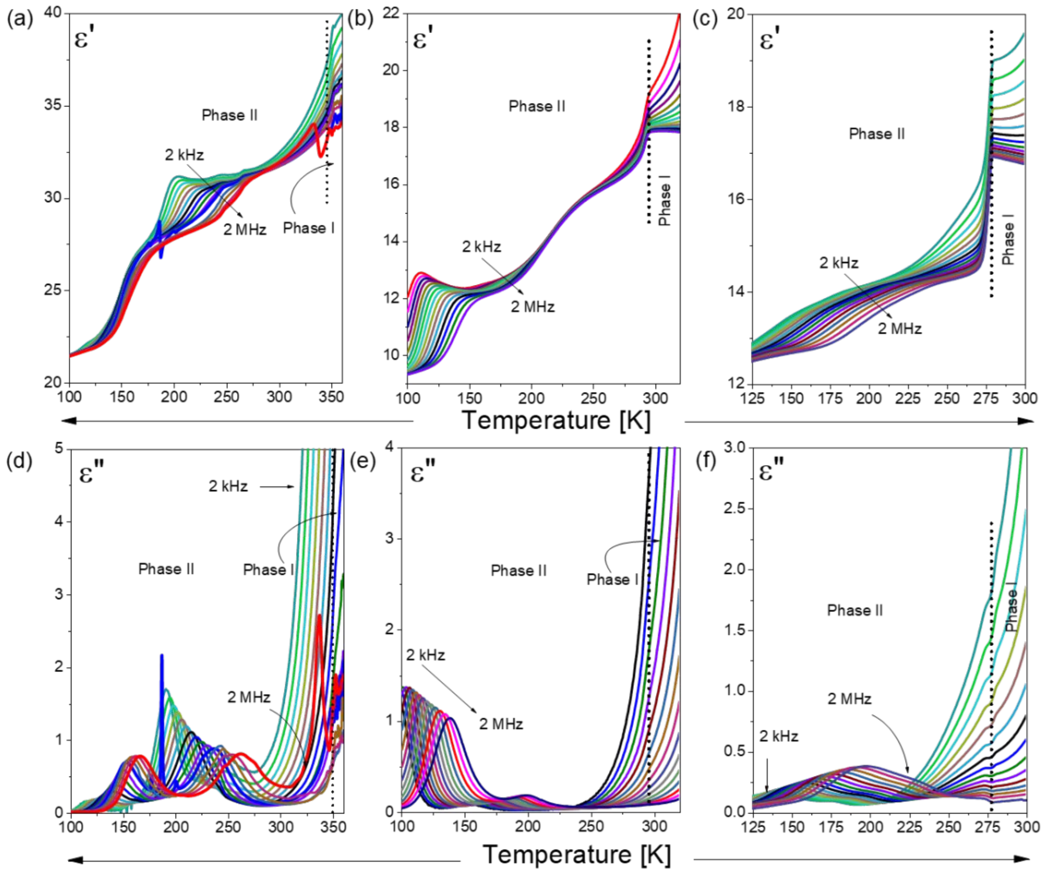

Figure S11. The temperature dependence of the complex dielectric permittivity for (a, d) **ACA** (single crystal/along *c*-axis), (b, e) **ABA** and (c, f) **AIA** (pellet samples) measured during heating cycle.

Section 6: Solid state static calculations

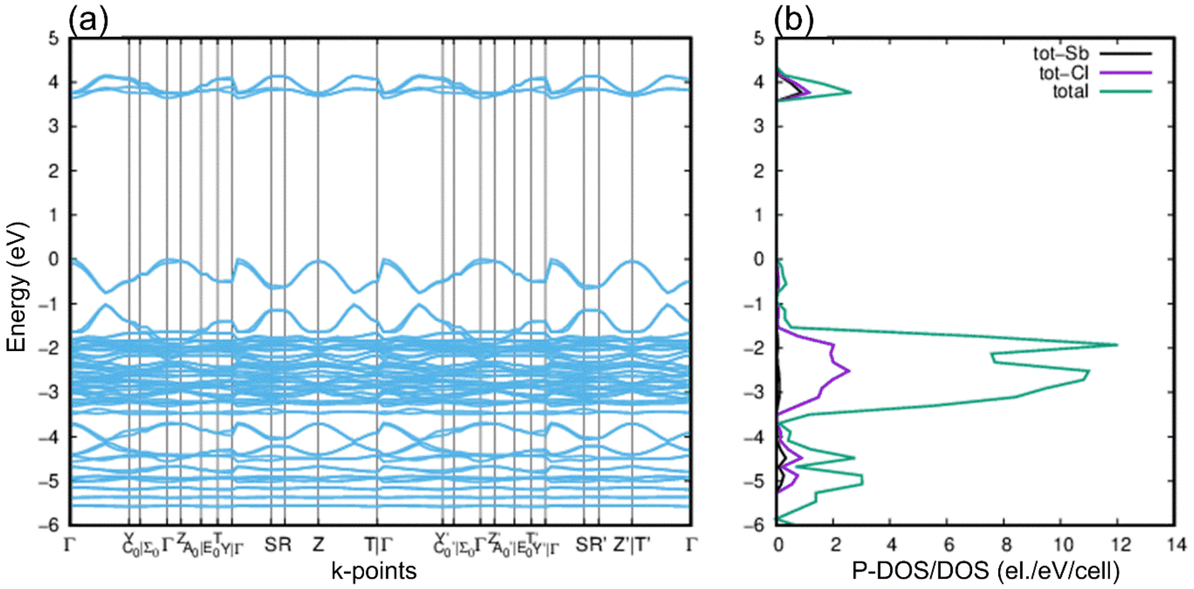

Figure S12. (a) The unified electronic band structure (EBS) E_g= 3.64 eV and (b) the density of states (DOS|P-DOS) of **ACA** crystal derived from the *ab initio* calculations.

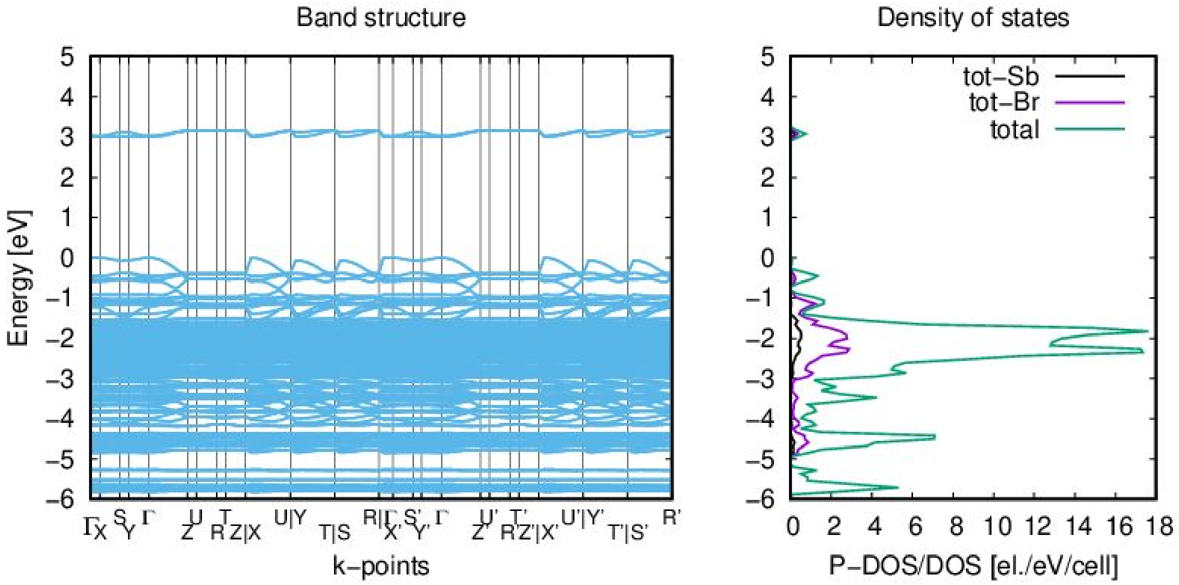

Figure S13. (a) The unified electronic band structure (EBS) E_g= 3.00 eV and (b) the density of states (DOS|P-DOS) of **ABA** crystal derived from the ab initio calculations.

Table S8 Geometric and lattice parameters of the **ACA** crystal after optimization at the DFT (HSE06-D3/pob_TZVP_rev2) method level compared with the experimental ones.

Bonds	Expt. [Å]	Calc. [Å]	Angles	Expt. [deg.]	Calc. [deg.]	Dihedrals	Expt. [deg.]	Calc. [deg.]
Sb1-Cl11	2.482	2.532	Cl11-Sb1-Cl12	90.76	90.30	Cl11-Sb1-Cl21-Sb2	-6.3	-6.4
Sb1-Cl12	2.467	2.511	Cl11-Sb1-Cl21	90.58	90.30	Cl12-Sb1-Cl21-Sb2	84.4	87.6
Sb1-Cl21	2.854	2.846	Cl11-Sb1-Cl22	84.22	86.85	Cl22-Sb1-Cl21-Sb2	-90.5	-90.4
Sb1-Cl22	2.987	2.869	Cl11-Sb1-Cl11	89.13	90.30	Cl11-Sb1-Cl21-Sb2	78.4	77.22
Sb1-Cl11	2.483	2.511	Cl11-Sb1-Cl21	176.90	171.02	Cl21-Sb1-Cl21-Sb2	170.6	171.5
Sb1-Cl21	2.854	2.846	Cl12-Sb1-Cl21	86.16	86.85	Cl11-Sb1-Cl21-Sb2	-78.4	-77.8
Sb2-Cl21	2.996	2.902	Cl12-Sb1-Cl22	172.95	177.14	Cl12-Sb1-Cl21-Sb2	-84.4	-85,78
Sb2-Cl31	2.431	2.481	Cl12-Sb1-Cl11	90.76	99.49	Cl21-Sb1-Cl21-Sb2	-170.6	-171.8
Sb2-Cl32	2.432	2.489	Cl12-Sb1-Cl21	86.16	83.37	Cl22-Sb1-Cl21-Sb2	90.5	90.78
Sb2-Cl32	2.432	2.489	Cl21-Sb1-Cl22	98.81	99.49	Cl11-Sb1-Cl21-Sb2	0.10	0.16
Sb2-Cl21	2.996	2.902	Cl21-Sb1-Cl11	176.91	175.01	Cl31-Sb2-Cl21-Sb1	96.1	98.02
Cl21-Sb2	2.996	3.002	Cl21-Sb1-Cl21	89.54	90.17	Cl32-Sb2-Cl21-Sb1	-172.8	171.9
N1A-H1AA	0.881	1.014	Cl22-Sb1-Cl11	84.22	85.82	Cl32-Sb2-Cl21-Sb1	139.3	142.34
N1A-H1AB	0.880	1.014	Cl22-Sb1-Cl21	98.81	97.01	Cl21-Sb2-Cl21-Sb1	10.0	11.7
N1A-C1A	1.308	1.314	Cl11-Sb1-Cl21	90.58	90.30	H1AA-N1A-C1A-C2A	-177.5	-179.30
C1A-C2A	1.493	1.476	Cl21-Sb2-Cl31	86.53	85.82	H1AA-N1A-C1A-N1A	0.0	0.02
C1A-N1A	1.308	1.314	Cl21-Sb2-Cl32	86.19	85.82	H1AB-N1A-C1A-C2A	1.5	1.54
C2A-H2AA	0.980	1.093	Cl21-Sb2-Cl32	176.41	175.01	H1AB-N1A-C1A-N1A	180.0	181.3
C2A-H2AB	0.980	1.080	Cl21-Sb2-Cl21	96.08	102.51	N1A-C1A-C2A-H2AA	89.0	86.78
C2A-H2AB	0.980	1.080	Cl31-Sb2-Cl32	90.85	90.85	N1A-C1A-C2A-H2AB	-31.0	32.44
N1A-H1AA	0.881	1.014	Cl31-Sb2-Cl32	90.85	90.87	N1A-C1A-C2A-H2AB	-151.0	-151.2
N1A-H1AB	0.880	1.014	Cl31-Sb2-Cl21	86.53	85.85	N1A-C1A-C2A-H2AA	-89.0	-90.2
N1B-H1BA	0.880	1.014	Cl32-Sb2-Cl32	91.40	91.07	N1A-C1A-C2A-H2AB	151.0	152.3
N1B-H1BB	0.879	1.015	Cl32-Sb2-Cl21	176.41	177.54	N1A-C1A-C2A-H2AB	31.0	32.7
N1B-C1B	1.308	1.311	Cl32-Sb2-Cl21	86.19	58.95	N1A-C1A-N1A-H1AA	-0.02	-0.08
C1B-C2B	1.483	1.476	Sb1-Cl21-Sb2	176.68	177.54	N1A-C1A-N1A-H1AB	-180.0	-181.23
C1B-N1B	1.308	1.311	Cl21-Sb2-Cl32	176.68	147.64	C2A-C1A-N1A-H1AA	177.5	176.8
C2B-H2BA	0.980	1.088	H1AA-N1A-H1AB	120.10	118.38	C2A-C1A-N1A-H1AB	-2.4	-2.78
C2B-H2BB	0.980	1.088	H1AA-N1A-C1A	120.00	119.06	H1BA-N1B-C1B-C2B	-179.4	-180.34
C2B-H2BB	0.980	1.088	H1AB-N1A-C1A	119.90	119.32	H1BA-N1B-C1B-N1B	-0.1	-0.02
N1B-H1BA	0.880	1.017	N1A-C1A-C2A	119.20	119.32	H1BB-N1B-C1B-C2B	0.6	-0.8
N1B-H1BB	0.879	1.017	N1A-C1A-N1A	121.60	122.27	H1BB-N1B-C1B-N1B	180.0	181.9
N1C-H1CA	0.881	1.017	C2A-C1A-N1A	119.20	119.06	N1B-C1B-C2B-H2BA	-90.0	-90.0
N1C-H1CB	0.880	1.017	C1A-C2A-H2AA	108.00	108.54	N1B-C1B-C2B-H2BB	155.0	157.4
N1C-C1C	1.299	1.313	C1A-C2A-H2AB	110.00	110.72	N1B-C1B-C2B-H2BB	24.0	24.34
C1C-C2C	1.485	1.479	C1A-C2A-H2AB	110.00	108.30	N1B-C1B-C2B-H2BA	90.0	90.30
C1C-N1C	1.299	1.301	H2AA-C2A-H2AB	110.00	108.30	N1B-C1B-C2B-H2BB	-24.0	-25.0
C2C-H2CA	0.980	1.088	H2AA-C2A-H2AB	110.00	108.30	N1B-C1B-C2B-H2BB	-155.0	-155.8
C2C-H2CB	0.980	1.088	H2AB-C2A-H2AB	109.00	110.20	N1B-C1B-N1B-H1BA	0.10	-0.23
C2C-H2CB	0.980	1.088	C1A-N1A-H1AA	120.00	118.38	N1B-C1B-N1B-H1BB	-180.0	-181.3
N1C-H1CA	0.881	1.017	C1A-N1A-H1AB	119.90	119.32	C2B-C1B-N1B-H1BA	179.4	179.0
N1C-H1CB	0.880	1.017	H1AA-N1A-H1AB	120.10	119.91	C2B-C1B-N1B-H1BB	-0.6	-0.68
			H1BA-N1B-H1BB	120.10	119.91	H1CA-N1C-C1C-C2C	-178.5	-176.55
			H1BA-N1B-C1B	119.90	122.53	H1CA-N1C-C1C-N1C	-0.0	-0.03
			H1BB-N1B-C1B	120.00	122.53	H1CB-N1C-C1C-C2C	1.5	1.55

**Cell [Å]**	**Expt.**	**Calc.**	N1B-C1B-C2B	119.60	120.32	H1CB-N1C-C1C-N1C	-180.0	-179.09
a	8.476	8.354	N1B-C1B-N1B	120.80	122.23	N1C-C1C-C2C-H2CA	89.0	87.87
b	16.087	15.866	C2B-C1B-N1B	119.60	118.78	N1C-C1C-C2C-H2CB	-150.0	-152.3
c	17.691	17.127	C1B-C2B-H2BA	98.00	99.20	N1C-C1C-C2C-H2CB	-31.0	-31.22
**Angles [deg.]**	**Expt.**	**Calc.**	C1B-C2B-H2BB	118.00	119.38	N1C-C1C-C2C-H2CA	-89.0	-89.3
α	90.00	90.00	C1B-C2B-H2BB	118.00	119.38	N1C-C1C-C2C-H2CB	31.0	32.4
β	90.00	90.00	H2BA-C2B-H2BB	107.00	108.72	N1C-C1C-C2C-H2CB	150.0	150.4
γ	90.00	90.00	H2BA-C2B-H2BB	107.00	108.72	N1C-C1C-N1C-H1CA	0.0	0.00
			H2BB-C2B-H2BB	107.00	108.72	N1C-C1C-N1C-H1CB	180.0	181.2
			C1B-N1B-H1BA	119.90	119.32	C2C-C1C-N1C-H1CA	178.5	178.36
			C1B-N1B-H1BB	120.00	122.27	C2C-C1C-N1C-H1CB	-1.5	-1.75
			H1BA-N1B-H1BB	120.10	122.27			
			H1CA-N1C-H1CB	120.00	122.27			
			H1CA-N1C-C1C	120.00	122.27			
			H1CB-N1C-C1C	120.00	122.27			
			N1C-C1C-C2C	119.70	119.16			
			N1C-C1C-N1C	120.50	122.23			
			C2C-C1C-N1C	119.70	119.16			
			C1C-C2C-H2CA	112.00	114.23			
			C1C-C2C-H2CB	107.00	108.03			
			C1C-C2C-H2CB	107.00	108.03			
			H2CA-C2C-H2CB	110.00	111.16			
			H2CA-C2C-H2CB	110.00	111.16			
			H2CB-C2C-H2CB	110.00	111.16			
			C1C-N1C-H1CA	120.00	122.72			
			C1C-N1C-H1CB	120.00	122.72			
			H1CA-N1C-H1CB	120.00	122.27			

Table S9. Geometric and lattice parameters of the **ABA** crystal after optimization at the DFT (HSE06 D3/pob_TZVP_rev2) method level compared with the experimental ones. Symmetry code from table S6.

Bonds	Expt. [Å]	Calc. [Å]	Angles	Expt. [deg.]	Calc. [deg.]	Cell [Å]	Expt. [deg.]	Calc. [deg.]
Sb1-Br11	3.056	3.076	Br11-Sb1-Br12	98.30	99.23	a	8.630	8.592
Sb1-Br11	3.056	3.076	Br21-Sb1-Br11	86.52	85.33	b	16.718	16.693
Sb1-Br12	2.162	2.172	Br21-Sb1-Br12	173.15	176.54	c	37.493	37.946
Sb1-Br21	2.604	2.632	Br22-Sb1-Br11	89.39	92.03	**Angles [deg]**		
Sb1-Br22	2.613	2.615	Br22-Sb1-Br12	83.95	84.52	α	90.0	90.0
Sb1-Br22	2.613	2.615	Br22-Sb1-Br11	177.70	178.52	β	90.0	90.0
Sb2-Br31	2.631	2.641	Br22-Sb1-Br12	83.95	84.35	γ	90.0	90.0
Sb2-Br31	2.631	2.641	Br22-Sb1-Br22	91.31	91.45			
Sb2-Br32	2.615	2.625	Br21-Sb1-Br22	91.28	91.52			
Sb2-Br41	3.077	3.082	Br21-Sb1-Br22	91.28	92.15			
Sb2-Br42	3.045	3.047	Br31-Sb2-Br31	93.37	93.47			
Sb2-Br42	3.045	3.046	Br32-Sb2-Br31	94.07	95.25			
			Br32-Sb2-Br31	94.07	95.25			
			Br31-Sb2-Br41	87.87	86.32			
			Br31-Sb2-Br41	87.87	86.40			
			Br31-Sb2-Br41	91.68	92.32			

Br31-Sb2-Br41	91.68	92.33
Br31-Sb2-Br41	173.20	174.12
Br31-Sb2-Br42	173.20	174.12
Br32-Sb2-Br41	177.16	178.65
Br32-Sb2-Br42	90.10	90.20
Br32-Sb2-Br42	90.10	90.20
Br42-Sb2-Br41	87.77	88.23
Br42-Sb2-Br41	87.77	88.23
Br42-Sb2-Br42	82.94	83.54
Sb2-Br42-Sb2	76.13	78.22

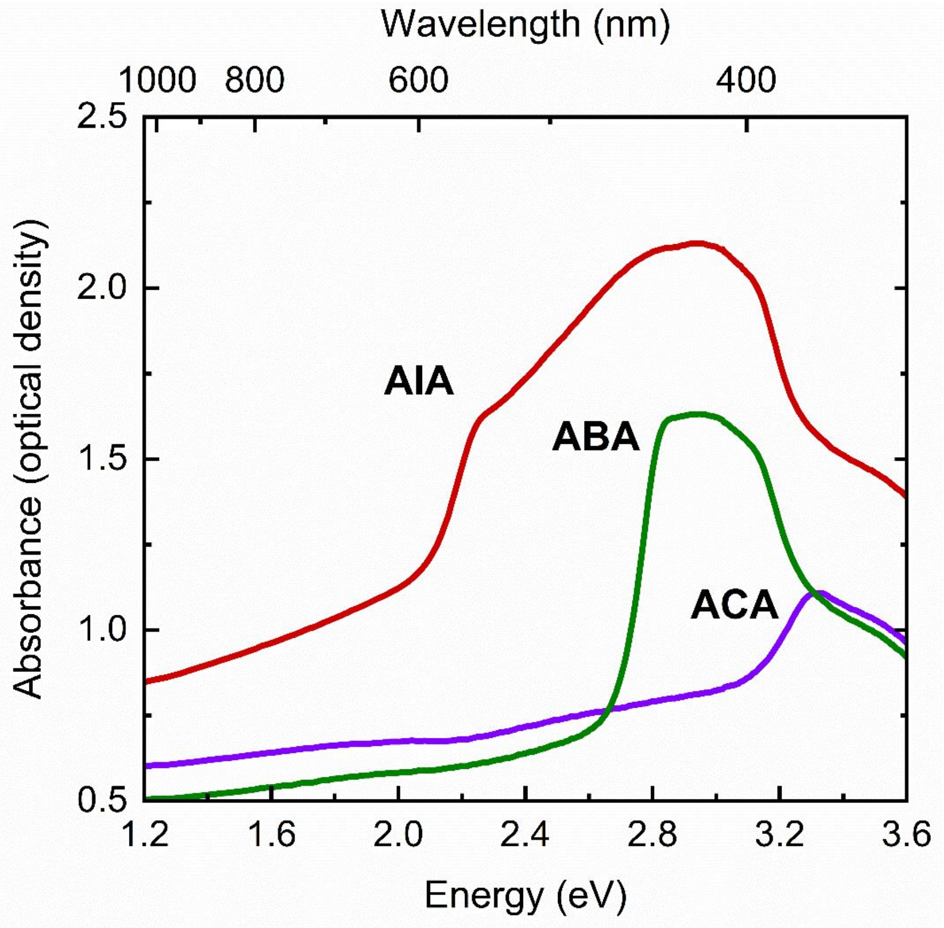

Figure S14. Optical absorption spectra of **AIA**, **ABA**, and **ACA**.

Section 7: Group theoretical description of domain patterns.

The scheme of the mutual orientation of domains and stress-free domain boundaries for the ferroic species *6/mmmFmmm* is depicted in Figure S15. Black lines correspond to the limit of vanishing spontaneous strain, and the green ones to a finite spontaneous strain. The construction of the scheme is analogous to that of work.[1] The angles $\alpha_1 \ldots \alpha_4$ also reported in the scheme are given by the following formulae.

$$\alpha_1 = 2arctan\left(\frac{a}{b}\right) + arctan\left(\frac{b}{a}\right) - arctan\left(\frac{b}{3a}\right) \ , \qquad \text{(S1)}$$

$$\alpha_2 = arctan\left(\frac{b}{a}\right) - arctan\left(\frac{b}{3a}\right) \ , \qquad \text{(S2)}$$

$$\alpha_3 = arctan\left(\frac{b}{a}\right) + arctan\left(\frac{b}{3a}\right) \ , \qquad \text{(S3)}$$

$$\alpha_4 = 2arctan\left(\frac{a}{b}\right) + arctan\left(\frac{b}{a}\right) + arctan\left(\frac{b}{3a}\right) \ . \qquad \text{(S4)}$$

For completeness, we quote the angles w_m and w_r by which a domain should be rotated with respect to the neighbouring domain to form a W and W' wall, respectively[2] in the presence of finite spontaneous strain.

$$w_m = \left(\frac{\pi}{3}\right) - 2arctan\left(\frac{b}{3a}\right) \ , \qquad \text{(S5)}$$

$$w_r = 2\,arctan\left(\frac{a}{b}\right) - \left(\frac{\pi}{3}\right) \ . \qquad \text{(S6)}$$

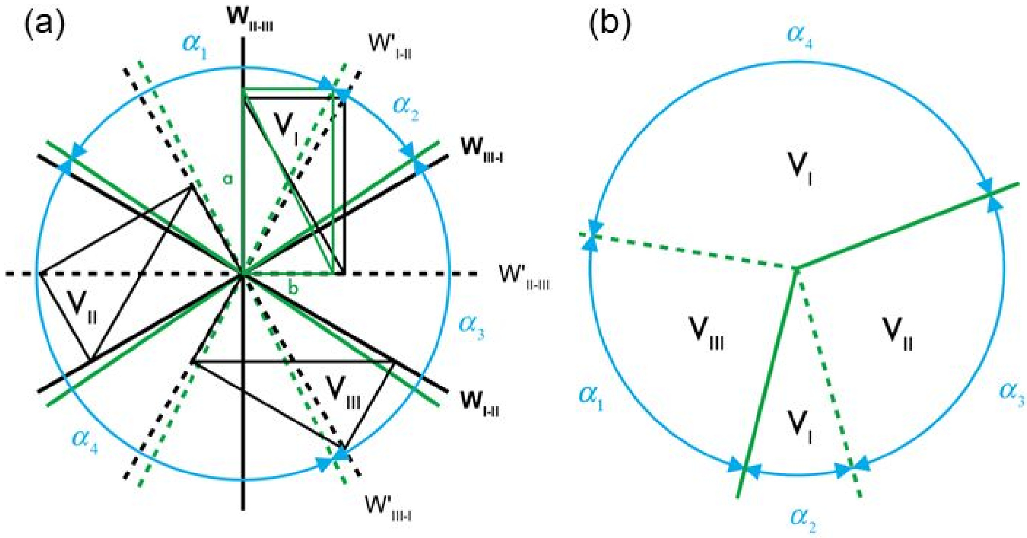

Figure S15. (a) Scheme of ferroelastic domains V_I, V_{II} and V_{III} and their boundaries. Black lines correspond to the limit of vanishing strain. Green lines show the traces of domain boundaries bordering domain V_I (also sketched in green) in the deformed system. Lengths *a* and *b* are lattice constants in the orthorhombic deformed phase. The W domain boundaries along the mirror planes lost in the symmetry reduction are marked with bold solid lines and the W' boundaries with dashed lines. The angles $\alpha_1 \ldots \alpha_4$ can be calculated with eqs. (S1) –(S4). (b) W and W' domain walls forming the node (3,1,3,5) with the angles $\alpha_1 \ldots \alpha_4$ marked.[3] To better follow the correspondence with the angles from Figure 15(a) one should realize that the sector α_2 of the domain V_I corresponds to the analogous sector in Figure 15(b).

The angles visualized in Figure S15(a) should be present in the stress-free node schematized in Figure S15(b). This node is labeled (3,1,3,5) in [3] where the figures in parentheses refer to the number of sectors, which in the zero-strain limit amount to 30°. This is the only configuration among 42 possible intersection/branching points that allows all three domains to meet at a single point and to rest, nevertheless, unstrained. The values of angles predicted by eqs. (S1 – S4) are expected for an isolated (3,1,3,5) node in an unstressed homogeneous sample. All other nodes and intersection points require some deformation of certain domains, which deformations were referred to as disclinations.[4] The unique node (3,1,3,5) is sketched in Figure S15(b). The spontaneous strain can be calculated from lattice constants obtained via X-ray diffraction (XRD) experiments. This strain varies with temperature. Moreover, because of symmetry relations, it must undergo a discontinuity at the PT so that the limit of vanishing strain, i.e. $b/a \rightarrow \sqrt{3}$, is unlikely to be exactly achieved in experiments. For any ratio *b/a* the angles between the domain boundaries follow from eqs. (S1)-(S4). The angles marked as $\alpha_I \ldots \alpha_4$ in Figures S7(a-b) and calculated with the present XRD data, are gathered in Table S10. The structure of the

LT phase of **AIA** is not precisely determined and has been tentatively attributed to a triclinic system with slight deviations from the initial hexagonal structure. These discrepancies may be due to a very fine domain pattern and/or an interplay with possible stacking faults due to a very long lattice parameter c. In Table S10 we reported data for this averaged triclinic structure, selecting the unit cell in analogy with Figure S14(a).

Table S10. The values of angles marked in Figures S15(a-b) and given in eqs. (S1-S6) predicted for the crystals under study from crystallographic data. In the last row, we give the angle of disclination characteristic for the star pattern.[2] The asterisk indicates a possibly averaged structure of **AIA** with parameters from Figure S8. Uncertainties are deduced only from the accuracy of XRD data to allow the reader to judge the influence of stresses in samples.

	ACA	ACA	ABA	AIA
T (K)	295	100	100	100
b/a	1.806(1)	1.898(1)	1.937(1)	1.732(2) *
α1	87.92(3)°	85.46(3)°	84.45(3)°	89.99(1)°
α2	29.9783(7)°	29.896(2)°	29.845(1)°	29.999999(1)°
α3	92.077(9)°	94.535(9)°	95.55(8)°	90.009(8)°
α4	150.0237(7)°	150.104(2)°	150.155(2)°	150.000000(1)°
w_m	-2.10(3)°	-4.64(3)°	-5.70(3)°	-0.01(1)°
w_r	-2.06(3)°	-4.43(3)°	-5.39(9)°	-0.01(1)°
$3w_m$ - $3w_r$	-0.13(28)°	-0.6(2)°	-0.9(2)°	0.00(6)°

Figure S7 suggests six ferroelastic domains in **ABA** instead of three. Three explanations are in principle possible: (i) twinning of the crystal occurring already in the high-symmetry phase; (ii) the low-symmetry phase of **ABA** being monoclinic rather than orthorhombic; or (iii) angular deviations of domains due to disclinations arising at nodes with disclinations are spuriously attributed to lower symmetry. We argue that the third possibility is most plausible. A hypothesis of a monoclinic distortion was also stated in ref.[5] for a chemically simpler system. Future local structural measurements may settle which of these hypotheses actually holds. A new property of the star pattern in **ABA** is that the particular stars are sometimes parallel or

perpendicular to each other. This can be seen in Figure S16 where the star rays (wedges) of the same colour are apparently either parallel or perpendicular. Figure S17 is an example in which two different ferroelastic domains have apparently the same colour (cyan). The origin of this effect is still to be elucidated.

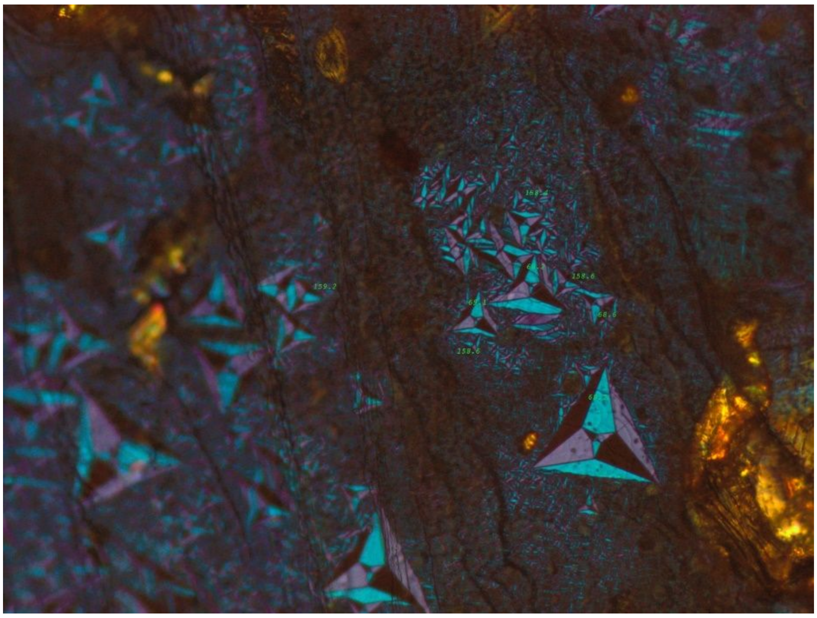

Figure S16. Parallel and perpendicular stars in **ABA**. The wedges (rays) of the same colour are parallel and perpendicular up to 0.2°.

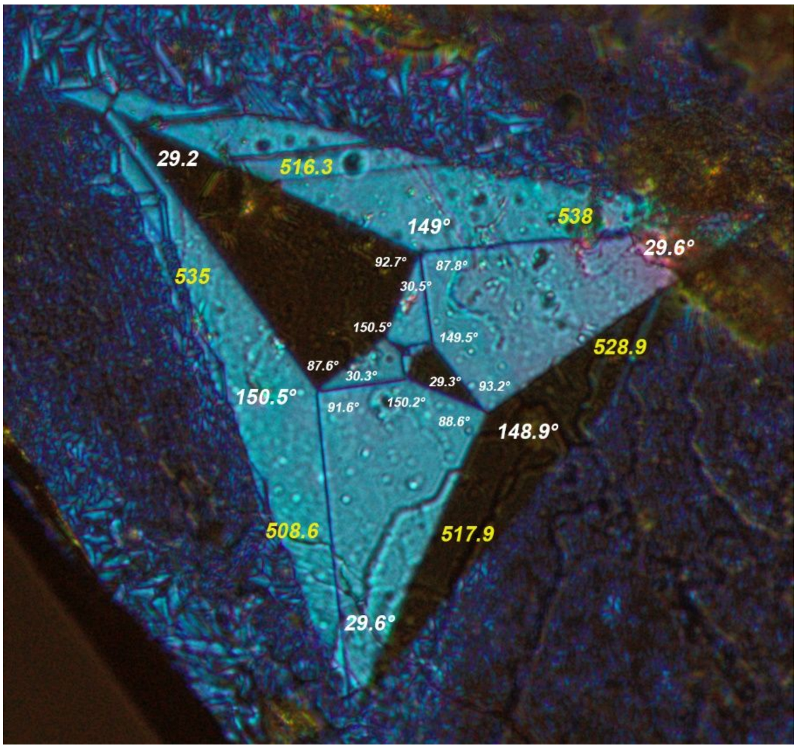

Figure S17. A two-colour star in **ABA** (T = 270 K). Experiment with rotated sample and/or light beam may reveal if this is a result of two domains being subtending equal angles with the polarization vector of the light.

Figure S18 is an attempt to ascribe particular domains to the optical image of the stressed ferroelastic texture of **ACA** presented in Figure 8(f) under the assumption that the only allowed angles between the domain walls are those of Figure S15(a).

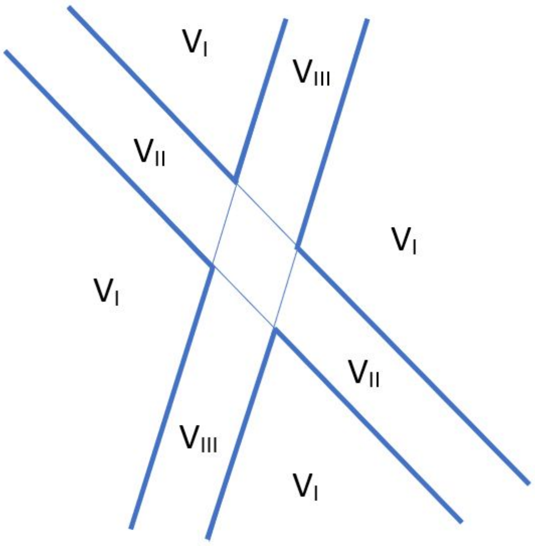

Figure S18. Scheme of a typical node of the stressed part of Figure 8(f) with tentative attribution of ferroelastic variants to particular regions.

References

(1) Szklarz, P.; Pietraszko, A.; Jakubas, R.; Bator, G.; Zieliński, P.; Gałazka, M. Structure, Phase Transitions and Molecular Dynamics of $[C(NH_2)_3]_3[M_2I_9]$, M = Sb, Bi. *J. Phys: Condens. Matter* **2008**, *20* (25), 255221. https://doi.org/10.1088/0953-8984/20/25/255221.

(2) Kitano, Y.; Kifune, K. HREM Study of Disclinations in MgCd Ordered Alloy. *Ultramicroscopy* **1991**, *39* (1–4), 279–286. https://doi.org/10.1016/0304-3991(91)90207-M.

(3) Curnoe, S. H.; Jacobs, A. E. Statics and Dynamics of Domain Patterns in Hexagonal-Orthorhombic Ferroelastics. *Phys. Rev. B* **2001**, *63* (9), 094110. https://doi.org/10.1103/PhysRevB.63.094110.

(4) Kitano, Y.; Kifune, K.; Komura, Y. Star Lisclination in a ferro-elastic material B19 MgCd alloy. *J. Phys. Colloques* **1988**, *49* (C5), C5-201-C5-206. https://doi.org/10.1051/jphyscol:1988520.

(5) Vicens, J.; Delavignette, P. A Particular Domain Configuration Observed in a New Phase of the Ta-N System. *Phys. Status Solidi (a)* **1976**, *33* (2), 497–509. https://doi.org/10.1002/pssa.2210330208.
